# Quantitative Determination and Environmental Risk Assessment of 102 Chemicals of Emerging Concern in Wastewater-Impacted Rivers Using Rapid Direct-Injection Liquid Chromatography—Tandem Mass Spectrometry

**DOI:** 10.3390/molecules26185431

**Published:** 2021-09-07

**Authors:** Melanie Egli, Alicia Hartmann, Helena Rapp Wright, Keng Tiong Ng, Frédéric B. Piel, Leon P. Barron

**Affiliations:** 1 Medical Research Council Centre for Environment and Health, School of Public Health, Faculty of Medicine, Imperial College London, 86 Wood Lane, London W12 0BZ, UK; m.egli20@imperial.ac.uk (M.E.); hartmann.alicia@stud.hs-fresenius.de (A.H.); h.rapp-wright@imperial.ac.uk (H.R.W.); keng_tiong.ng@imperial.ac.uk (K.T.N.); f.piel@imperial.ac.uk (F.B.P.); 2 Hochschule Fresenius gem. GmbH, Limburger Str. 2, 65510 Idstein, Germany

**Keywords:** direct injection analysis, pharmaceuticals, pesticides, river water, wastewater, rapid analysis

## Abstract

The rapid source identification and environmental risk assessment (ERA) of hundreds of chemicals of emerging concern (CECs) in river water represent a significant analytical challenge. Herein, a potential solution involving a rapid direct-injection liquid chromatography–tandem mass spectrometry method for the quantitative determination of 102 CECs (151 qualitatively) in river water is presented and applied across six rivers in Germany and Switzerland at high spatial resolution. The method required an injection volume of only 10 µL of filtered sample, with a runtime of 5.5 min including re-equilibration with >10 datapoints per peak per transition (mostly 2 per compound), and 36 stable isotope-labelled standards. Performance was excellent from the low ng/L to µg/L concentration level, with 260 injections possible in any 24 h period. The method was applied in three separate campaigns focusing on the ERA of rivers impacted by wastewater effluent discharges (1 urban area in the Basel city region with 4 rivers, as well as 1 semi-rural and 1 rural area, each focusing on 1 river). Between 25 and 40 compounds were quantified directly in each campaign, and in all cases small tributary rivers showed higher CEC concentrations (e.g., up to ~4000 ng/L in total in the R. Schwarzach, Bavaria, Germany). The source of selected CECs could also be identified and differentiated from other sources at pre- and post- wastewater treatment plant effluent discharge points, as well as the effect of dilution downstream, which occurred over very short distances in all cases. Lastly, ERA for 41 CECs was performed at specific impacted sites, with risk quotients (RQs) at 1 or more sites estimated as high risk (RQ > 10) for 1 pharmaceutical (diclofenac), medium risk (RQ of 1–10) for 3 CECs (carbamazepine, venlafaxine, and sulfamethoxazole), and low risk (RQ = 0.1–1.0) for 7 CECs (i.e., RQ > 0.1 for 11 CECs in total). The application of high-throughput methods like this could enable a better understanding of the risks of CECs, especially in low flow/volume tributary rivers at scale and with high resolution.

## 1. Introduction

The contamination of the environment with chemicals is now well established. Worldwide, ~350,000 chemical substances are licensed for manufacture and sale, and numerous new compounds are introduced every year [1]. In addition to these substances, transformation and metabolism to a suite of other compounds are also possible. The impacts of chemical pollution and the release of ‘novel entities’ to the environment are now being regarded as one of nine variables or ‘planetary boundaries’ for sustainable human life on Earth [2]. The planetary boundary threshold for novel entities is relatively unquantified and is increasingly being regarded as the third environmental crisis behind biodiversity loss and climate change [3].

With respect to the aquatic environment, a significant analytical challenge exists for the chemical characterisation, measurement, and environmental risk assessment (ERA) of many compounds with high spatiotemporal coverage. Multiresidue analysis methods currently exist for hundreds to thousands of compounds in water samples [4], but these often rely on analyte concentration steps such as solid phase extraction (SPE) to achieve the required sensitivity. For chemicals of emerging concern (CECs), this can prove challenging in terms of achieving sufficient chemical selectivity to ensure the widest range of compounds present in a sample can be extracted and enriched for instrumental measurement. Such pre-treatment steps bring throughput challenges for large-scale monitoring as they often require significant extra time, solvent consumption, labour, and cost. Online pre-treatment methods, including SPE coupled to liquid chromatography-mass spectrometry (LC-MS), for example, have been shown to increase throughput to some degree [5]. However, these can still suffer limited selectivity for application to large numbers of chemically diverse CECs as well as the potential for analyte and matrix carryover if the methods are not properly optimised, especially in complex samples. While direct-injection MS methods are still not widely considered for the quantitation of large numbers of CECs due to the potential for significant matrix effects, recently, direct-injection LC-MS of CECs in water has emerged, usually with gradient pre-separation to minimise these effects [6,7,8,9,10,11,12,13,14]. In many cases, this requires the large volume injection (LVI) of sample (typically between 80–5000 µL) and can help overcome the issue of analyte selectivity loss during pre-treatment. For example, Wilkinson et al. recently reported the use of LVI of 100 µL of sample onto a 33.1-min LC-MS/MS method for 61 pharmaceuticals in surface water and wastewater, amongst others, and offered promising performance in line with other multiresidue LVI methods reported in the literature [15,16,17]. With the general requirement for smaller sample sizes, this enabled more convenient international shipment and stability assessment of samples in transit.

Though a very attractive solution, LVI methods can be problematic when applied to long batch sequences where, arguably, contamination of electrospray sources in LC-MS based methods can occur and where regular ESI source cleaning is required. Martínez Bueno et al. addressed this issue by developing a direct-injection method using only 10 µL volumes in a 26-min LC-MS run (including re-equilibration) with lower limits of quantification (LLOQs) between 10 and 700 ng/L for wastewater and 0.5–700 ng/L for river water [18]. Albergamo et al. also developed a direct-injection high-resolution accurate mass spectrometry (HRMS) method for the determination of 33 CECs using only 30–40 µL injection volumes onto a biphenyl LC stationary phase, which also opened the potential for suspect screening of more CEC-related compounds to be performed [19]. Biphenyl phases offer many advantages over octadecylsilica, including different capacity and selectivity for aromatic, polar, and ionizable compounds. However, at least for current direct-injection methods for CECs, the potential for shorter gradient analysis seems underexplored to make this more scalable for higher spatiotemporal resolution monitoring campaigns. Notable recent progress was made by Couchman et al., who successfully separated 20 compounds in 36 s in a clinical toxicology application [20]. Recently, we attempted to translate this approach for rapid direct-injection analysis of 135 CECs in influent wastewater using a short 5 × 3 mm biphenyl column which only required small sample volumes with a runtime of 5.5 min including re-equilibration [8]. Smaller injection volumes (10 µL) and standard flow rates (0.5 mL/min) enabled very stable measurement performance over longer batches of samples. We subsequently evaluated its quantitative performance for 33 CECs as part of a small monitoring campaign in brackish river water (River Thames, UK) and passive sampler extracts [7]. The method yielded promising performance for this small set of CECs with detection limits at 3–8 ng/L and excellent linearity overall.

The aim of this work was to assess the broader performance and application of this rapid direct-injection analytical method for freshwater river monitoring using standard LC-MS/MS injection volumes. The objectives were: (a) to assess method performance of a larger number of CECs in freshwater matrix; (b) to better understand the impact of sample filtration on recovery; (c) to apply the method in three high spatial resolution campaigns in Germany and Switzerland, covering a variety of rural, semi-rural, and urban locations; and (d) to perform rapid ERA for quantifiable compounds through calculation of risk quotients. Ultimately, this work is the first to apply such a fast method using a very small sample volume which could potentially be used for large CEC monitoring and ERA campaigns.

## 2. Experimental

### 2.1. Reagents and Materials

HPLC grade methanol (Dorset, UK), HPLC-grade acetonitrile (Rehovot, Israel), hydrochloric acid (37% *v*:*v*) (Steinheim, Germany), and formic acid (Steinheim, Germany) were acquired from Sigma-Aldrich. The Milli-Q water purification system (Millipore, Bedford, MA, USA) was used to generate ultrapure water (resistance of 18.3 MΩ cm). All reference materials and stable isotope-labelled internal standards were of ≥97% purity, and a full list can be found in the SI. All standards (either 1.0 mg/mL or 0.1 mg/mL) were prepared in methanol (Sigma-Aldrich, Steinheim, Germany) and stored in 20 mL clear silanised glass vials with solid closed top septa closure (Fisher Scientific, Germany). Furthermore, 30 mL Nalgene^TM^ Narrow-Mouth LDPE bottles with screw-capped tops were used for sampling and were obtained from Thermo Scientific (Waltham, MA, USA). Safe-Lock tubes needed during sample preparation were ordered from Eppendorf (Hamburg, Germany) and 1.5 mL silanised amber glass vials with crimp neck were acquired from Fisher Scientific (Loughborough, UK). Whatman^TM^ 0.2 μm PTFE membrane filters (GE Healthcare Life Science, Little Chalfont, UK) and 1 mL Plastipak^TM^ syringes (BD, Berkshire, UK) were used for sample pre-treatment. 

### 2.2. Sampling Locations and Procedures

A total of 3 grab sampling campaigns were undertaken at 2 sites in Germany and 1 in Switzerland. The criteria for selection of all points in all campaigns included the ease of access to the riverbank for sampling, as well as points above and below the location of 1 or more WWTP discharge points and/or the location of tributary confluence points. All river water samples were taken as grab samples in 15–30 mL polyethylene bottles (Thermo Fisher Scientific, Waltham, MA, USA) which were previously rinsed with triplicate successive rinses with methanol, ultrapure water, and then river/wastewater at the site of sampling. Grab samples were all taken >0.5 m below the water surface in all cases either at the riverbanks directly or from food-grade polypropylene buckets which were cast and sunk into the river (~10–20 m distance) or from bridges with rope attached (buckets were rinsed thrice each time with river water before samples were taken). Samples were transferred to an ice-cold thermal bag and then frozen at −20 °C as soon as possible. This has generally been shown to preserve analyte stability [21,22]. However, no pH adjustment could be made prior to freezing at site, and some degree of microbial transformation could not be ruled out, though this would likely be low given that analysis was performed within only 2–3 weeks after sampling. Samples were transported by air to the London laboratory in a thermal bag and remained largely frozen upon arrival (total transit time of ~5–6 h in all cases).

Campaign 1 on the 23 August 2019 involved sampling across 10 locations (SZ1-10) along the River Schwarzach, Bavaria, Germany, in the Schwarzenbruck district between 10:00 and 18:00 h, covering a total distance of 2.5 km. This is a small river with low flow and volume. All sampling locations along this river were surrounded by thick forestry, but a wastewater treatment plant (WWTP) serving a local population of ~8500 discharges treated wastewater between locations SZ8 and SZ9. Part of the daily load of this WWTP includes wastewater from Rummelsberg Hospital. It is one of the largest orthopaedic clinics in Germany [23]. Every year 9000 inpatients (335 hospital beds) and 21,000 outpatients are treated there.

Campaign 2 was undertaken on the 3 January 2020 between 13:00 and 16:00 h along the River Emsbach in the Selters community (Hesse), with a similarly sized local population of ~8200. This is also a rural area surrounded by woods, meadows, and fields at all sampling sites. A total of 3 samples each were taken at 12 different sites (EM1-12) along a more extended route of ~12.5 km. A WWTP serving ~45,000 people discharges treated wastewater at EM7 [24]. This WWTP catchment area includes the community of Selters (Taunus) and the entire city of Bad Camberg with all its localities, as well as the villages of Waldorf, Heftrich, and Oberems. It also includes a medical park specialising in neurology, and the Median Group, specialising in orthopaedics and psychosomatics [25,26]. Samples near the WWTP effluent discharge were taken comparatively close to each other (~20–50 m) to help understand the dilution process in the river. No rainfall was recorded on the days prior sampling at either of the two German sites. Four replicate grab samples of each of wastewater influent and effluent were also taken at the WWTP, after the fine screen and the secondary clarifier, respectively. No further dilution took place between these 2 units.

Campaign 3 was carried out on the 4 January 2021 from 09:00 to 16:00 h, but 1 sample at RH10 was collected at 13:30 h on 30 December 2020 prior to the New Year celebrations. Grab samples in this campaign were all collected at the riverbank of the R. Rhine and 3 of its tributaries, R. Birs, Birsig, and Wiese, covering approximately 27 km in total. Sites RH9 and 10 were located at the tri-border area between France, Germany, and Switzerland and where the R. Rhine leaves Switzerland. The investigated areas within Basel-Stadt (city) and Basel-Landschaft (country) include 5 WWTPs (all of which discharge within rivers where sampling took place here). Those works combined treat 53.2 million cubic metres of wastewater and cover a population of 950,000 people [27,28,29,30,31,32]. The combined Basel area (city and country) has ~30 WWTPs, with a strong chemical industry comprising multiple research facilities and production sites. As to be expected, several hospitals are also located in the city, with the University Hospital of Basel being the largest in Northwestern Switzerland.

### 2.3. Instrumentation

Liquid chromatography–tandem mass spectrometry was performed using an LCMS 8060 apparatus (Shimadzu Corporation, Kyoto, Japan). Separations were performed on a Shimadzu Nexera X2 ultra high-pressure LC apparatus (Shimadzu Corporation, Kyoto, Japan) configured with a short 5.0 × 3.0 mm, 2.7 μm particle size Raptor^TM^ biphenyl cartridge (Thames Restek, Saunderton, UK) housed within an EXP^®^ Direct Connect Holder. Multiple reaction monitoring (MRM) was performed with positive–negative mode switching, and quadrupoles Q1 and Q3 were set to unit resolution. Supplementary information on the MS parameters is included in Table S1. As a collision-induced dissociation gas, Pureshield argon (BOC Gases, Guildford, UK) was used. The generation of dry air and nitrogen was achieved using a Genius 1051 gas generator (Peak Scientific, Inchinnan, UK). The injection volume was 10 μL and acetonitrile was the autosampler wash solvent between injections. A flow rate of 0.5 mL/min was used for all analysis. The 2 mobile phases A (0.1% formic acid in ultra-pure water (*v:v*)) and B (0.1% formic acid in methanol:acetonitrile (1:1; *v*:*v*)) were used at optimised gradient elution conditions: 10% mobile phase B for 0.20 min; a linear ramp from 10–60% from 0.2–3.0 min; a step gradient from 60–100% at 3.0 min; and holding at 100% B for a further 1.0 min before returning to the initial conditions, with a re-equilibration time of 1.0 min. Subsequently there was a further needle wash and sample injection cycle time of 30 s, leading to a total time of 5.5 min. Lab Solutions (version 5.93, Shimadzu) and Lab Solutions Insight (version 3.2, Shimadzu) were used to acquire and process chromatographic data. Microsoft Excel 2010 (version 16.48) and RStudio (version 1.1.463, RStudio, Boston, MA, USA) running R 3.5.1 were used for further data evaluation.

### 2.4. Analytical Method Performance Assessment

As Campaign 1 was conducted first in the timeline, a pooled river water sample containing equal proportions of all 10 sites was prepared from this set of samples for matrix-matching and method performance assessment. The method was evaluated following the International Council for Harmonisation of Technical Requirements for Pharmaceuticals for Human Use (ICH) guidelines [33]. Metrics for evaluation included linearity, range, matrix effects, limit of detection (LOD), LLOQ, and precision. For linearity, a background-corrected matrix-matched calibration was performed (10–10,000 ng/L, N ≥ 10) using a log_10_-weighted regression. The method range was determined over a minimum of N ≥ 5 concentration levels. Where corresponding stable isotope-labelled internal standards (SIL-IS) were available, these were used at a 500 ng/L spiking level for quality control purposes and to generate calibration curves using peak area ratios. This selected SIL-IS concentration level is consistent with our previous application of this method to wastewater and represents a suitable mid-range, environmentally relevant concentration for CECs in general [34,35]. For all other compounds, matrix-matched calibration was performed using background-subtracted peak areas in pooled freshwater samples from Campaign 1. Precision was determined using matrix-matched standards at 250 ng/L (*n* = 6) and 2500 ng/L (*n* = 6). The LOD was calculated as 3.3 times the standard error of the intercept of the calibration line divided by the slope of the calibration curve. The LLOQ was assigned as 3 times the LOD. Matrix effects (ME) were expressed as the percentage coefficient of variation between the peak area of background-subtracted matrix-matched standards and standards of analytes in ultrapure water at 250 ng/L (*n* = 6) and 2500 ng/L (*n* = 6). Carryover was assessed as the percentage of peak areas detected in mobile phase blanks run immediately after injection of spiked river water at 500, 2500, and 10,000 ng/L for all analytes in Table S3 (SIL-IS at 500 ng/L). Stability of retention time and peak area was performed using SIL-IS data for the full batch of method performance assessment samples.

### 2.5. Sample Preparation and Quantification Procedures

Samples were thawed in an upright position, then shaken and placed on a flat surface for sedimentation. For samples containing significant suspended matter, samples were centrifuged at 4000 rpm for 10 min (Centrifuge 5810 R, Eppendorf, Hamburg, Germany) and the supernatant used for further preparation. Background corrected, matrix-matched calibrations were performed (5–10,000 ng/L, N ≥ 10) for quantification of all CECs for each sample location and matrix type separately using a composite prepared from samples from each site. Quality control (QC) standards were run before, during, and after every batch. For all calibrants and samples, a consistent dilution factor was employed using 9 parts sample and 1 part diluent containing either the required concentration of all analyte reference materials and SIL-IS in methanol. Calibrated positive displacement pipettes were used throughout this work. This mixture was then filtered using 1 mL Plastipak syringes (BD, Berkshire, UK) configured to Whatman 0.2 μm Teflon membrane filters (GE Healthcare Life Science, Little Chalfont, UK) into 1.5 mL silanised amber glass vials by/obtained from Fisher Scientific (Germany) before analysis with LC-MS/MS.

Where possible, two transitions were used to confirm the occurrence of an analyte together with its retention time [36]. The most intense transition was used for quantification, while the second transition served for confirmation. For background subtraction, peak areas resulting from the analysis of the unfortified sample were subtracted from peak areas in matrix-matched calibrants as required. For analytes with no corresponding SIL-IS available, peak areas were used directly to perform external matrix-matched calibration quantification. However, where a corresponding SIL-IS was available, the peak area ratio method was employed across the range using a constant SIL-IS concentration of 500 ng/L.

### 2.6. Environmental Risk Assessment Procedures

Risk quotients (RQs) were calculated for each quantified compound for each campaign at selected sites with high CEC occurrence at SZ9, EM7, and RH5 [37]. Although the highest concentrations in Campaign 3 were measured at BS1, this site was an engineered river channel rather than a natural ecosystem and was therefore excluded in favour of RH5. RQs were calculated as the ratio of the measured environmental concentration (MEC) and the predicted no-effect concentration (PNEC) according to Palma et al. [38]. Where possible, verified PNEC data were obtained from the literature [39,40], and the lowest value was used in RQ calculations or was otherwise derived using the NORMAN Ecotoxicology Database quantitative structure–activity relationship-based prediction tool [41]. Different thresholds of risk were applied depending on the RQ: <0.1 (insignificant risk); 0.1–1.0 (low risk); 1–10 (medium risk); and >10 (high risk).

## 3. Results and Discussion

### 3.1. Direct-Injection LC-MS/MS Throughput and Sample Volume

**Figure 1:**
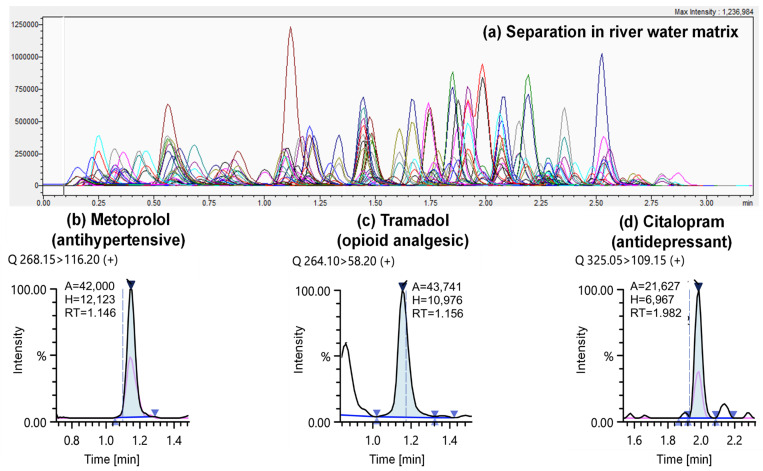
An example of LC-MS/MS separation of a 500 ng/L CEC mixed standard (**a**). Selected individual MRM chromatograms showing compounds quantified at low concentrations including (**b**) metoprolol (15 ng/L), (**c**) tramadol (11 ng/L), and (**d**) citalopram (11 ng/L) in river water samples taken in 2021 in Campaign 3.

For such a short column, excellent chromatographic selectivity was achieved in under 4 min for 164 compounds and 36 SIL-IS and using a simple gradient and standard flow rate (Figure 1a). Accounting for re-equilibration and autosampler cycle time, ~260 injections could be performed in a single 24-h period. Over 10 datapoints per peak were acquired for each transition for high-quality definition. Moreover, only 10 µL of sample was required for analysis (optimised). Other recent methods employing direct-injection LC-MS/MS have used up to 400 µL of sample [42], which could, for an extended analysis, potentially contaminate the ESI source or progressively deteriorate LC-MS/MS performance. In addition, this meant that large numbers of samples could be more conveniently shipped frozen to the London laboratory for analysis. While 15–30 mL bottles were used here, only 3 mL was sufficient for replicate preparations and for SPE if required. Recent work by Wilkinson et al. has shown that international shipment of samples over this timeframe generally resulted in no significant degradation [43] and this was also found in our previous work for wastewater shipments between Mexico, the United States, and the United Kingdom [8].

### 3.2. Method Performance Assessment

During an initial evaluation of performance, filtration recovery was assessed using ultrapure water and river water matrix at 100 ng/L. A total of 151 CECs and 26 SIL-IS could be reliably detected at this low concentration level in unfiltered standards. Mean recovery (±standard deviation, *n* = 3) for CECs following filtration was found to be 99 ± 35% and 86 ± 31% in ultrapure water and river water, respectively (Table S2) and the method was considered broadly suitable for CEC monitoring at this level. However, for some compounds recovery was very low. This was particularly the case for most macrolide antibiotics including some of those listed on the current EU watchlist, such as clarithromycin (748 Da, predicted logK_ow_ (ACD Labs) = 3.16), azithromycin (749 Da, predicted logK_ow_ = 3.33), spiramycin (843 Da, predicted logK_ow_ = 3.06), roxithromycin (837 Da, predicted logK_ow_ = 3.73), and josamycin (828 Da, logK_ow_ = 3.88). Importantly, this issue seemed to be matrix-dependent, and previous work on wastewater analysis did not reveal this problem for these compounds [8]. Conversely, recovery from river water for lincomycin was 114 ± 13 and 97 ± 1% here in ultrapure water and in river water, respectively (407 Da, predicted logK_ow_ = 0.91). In addition to these, early eluting compounds such as metformin were completely undetected in river water due to matrix suppression (extended column lengths and/or different stationary phases such as C_18_ or pentafluorophenyl could be evaluated to improve capacity and selectivity for such compounds, but likely at the cost of speed). Therefore, matrix complexity, molecular size, and/or hydrophobicity may explain this behaviour though it is unclear whether losses occurred via sorption to the cartridge housing or the membrane itself. No apparent losses in sensitivity were observed in non-filtered standard solutions prepared and stored using the same silanised laboratory glassware, vials, or containers. Therefore, where Teflon filters are used for such substances, it is recommended that an alternative material be used, or that centrifugation be used instead where possible. Importantly, filtration of river water should not be performed before addition of SIL-IS even if recovery is assumed to be low for a particular membrane material. Interestingly, the short 3 mm cartridge columns used in this study were far more cost-effective than longer analytical columns to replace if blockages occurred.

A deeper assessment method performance for 113 compounds was performed in matrix (Table 1). Of these, performance was considered satisfactory for 102 compounds at low–mid ng/L and environmentally relevant concentrations (Table S3). Linearity was excellent and almost all compounds displayed high coefficients for determination (R^2^ ≥ 0.99). Across all compounds, mean peak area imprecision (expressed as %RSD) was determined to be 10 (±15)% and 5 (±3)% at 250 and 2500 ng/L, respectively. Imprecision of ≥20% for 11 compounds was observed at 250 ng/L and for this reason, the method was considered semi-quantitative only for these compounds. Regarding sensitivity, the mean estimated LOD and LLOQ were 3 (±5) ng/L (median: 2 ng/L) and 9 (±17) ng/L (median: 5 ng/L). Standards were not prepared at these exact concentrations to verify LOQs, but calibrants were still included from 5 or 10 ng/L upwards in all subsequent applications to river water. Examples of MRMs for three selected low concentration level (≤15 ng/L) CECs found in river water in Campaign 3 are shown in Figure 1b–d. Though perhaps not as sensitive as methods employing SPE for preconcentration, this method included more compounds generally and was much faster than other direct-injection LC-MS/MS methods. On average, matrix effects at 250 ng/L and 2500 ng/L were also very low at ≤25%, which was considered excellent given that no sample clean-up was performed aside from filtration. Matrix effects at lower concentrations were not studied. The stability of retention time was excellent overall when examining all 36 SIL-IS across the 144-sample sequence (0.97 ± 1.61%) and no evidence of significant drift was observed (Figure S3 shows example data for MDMA-d5, benzoylecgonine-d3 and temazepam-d5 which eluted at the start, middle and end of the gradient, respectively). SIL-IS peak area %RSD was generally less than 15% with notable exceptions for those with corresponding non-labelled analogues which had low recovery following filtration (e.g., macrolide antibiotics). In addition, no major drift was observed in the river water analysis campaigns for both unlabelled compounds and SIL-IS (e.g., retention time and peak area %RSDs in 500 ng/L QC standards run at the start, middle, and end of the batch for Campaign 3 were 0.6 ± 0.6% and 9.0 ± 5.8%, respectively, over a total of 221 consecutive injections). Finally, carryover was very low for all compounds in Table S3 at 0.6, 0.9, and 0.2% in mobile phase blanks run immediately after pooled river water samples spiked at 500, 2500, and 10,000 ng/L, respectively (SIL-IS = 500 ng/L).

### 3.3. Occurrence of Emerging Contaminants in River Water

#### 3.3.1. Campaign 1: R. Schwarzach, Schwarzenbruck District, Germany

**Figure 2:**
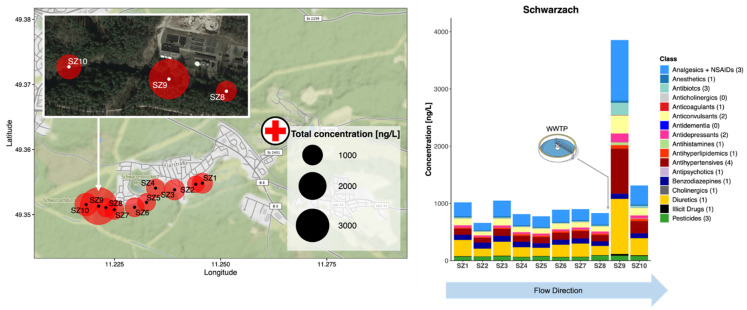
Occurrence of all CECs monitored in the River Schwarzach (SZ) in the Schwarzenbruck district, Bavaria, Germany, in grab samples taken in 2019. Red circles represent sampling points (labelled SZ1-10) and the size of the circle represents the combined total CEC concentration in ng/L. The hospital symbol represents the location of Rummelsberg Hospital. Individual compounds within each class can be found in Table S8.

In this small semi-rural catchment, 26 analytes were detected across all samples, of which 23 were quantified at least once (Figure 2 and Table S4). Across all sites, hydrochlorothiazide, diclofenac and carbamazepine accounted for 23%, 17%, and 9% of the total CEC concentrations measured, respectively. The WWTP discharge point was identifiable between sampling SZ8 and SZ9 by a 4-fold increase in combined analyte concentration to ~4000 ng/L. Of these, 21 were at least double in concentration at SZ9 in comparison to the average concentrations across SZ1-8 located above the WWTP. However, concentrations of any single compound across any site remained below 1000 ng/L on average (i.e., hydrochlorothiazide, a diuretic drug, at SZ9 showed the highest concentration measured, at 962 ± 78 ng/L). While concentrations increased at SZ9 generally, this was not the case for oxazepam (no change) and fenuron. For the latter, concentrations decreased by roughly one-third at SZ9, indicating that its primary source was unlikely from treated wastewater effluent. Recent wastewater data for large city sites in the United States, Mexico, and the United Kingdom did not determine fenuron above 250 ng/L, potentially supporting this hypothesis [8]. Fenuron however was detected at all sites in this river (range: 26–61 ng/L) and is an herbicide banned for use on crops within the EU since 2002 [35]. It is still manufactured as an additive in adhesives and sealants, coatings, fillers, putties, plasters, modelling clay, non-metal-surface treatment products, and polymers, and is commonly used in the building and construction industry. Therefore, its consistent occurrence across sites could be explained by continual release to the environment from such items as well as leaching from crops [44]. Fenuron has been measured by our group [7,8,45,46] and the Environment Agency in the United Kingdom in urban and rural rivers to varying degrees, and mostly at concentrations < 50 ng/L [47]. Total CEC concentrations fell by 3-fold at SZ10 ~200 m downstream. As there was a dam after the sampling site SZ10, no further samples were taken to understand any further dilution processes.

#### 3.3.2. Campaign 2: R. Emsbach, Selters, Germany

**Figure 3:**
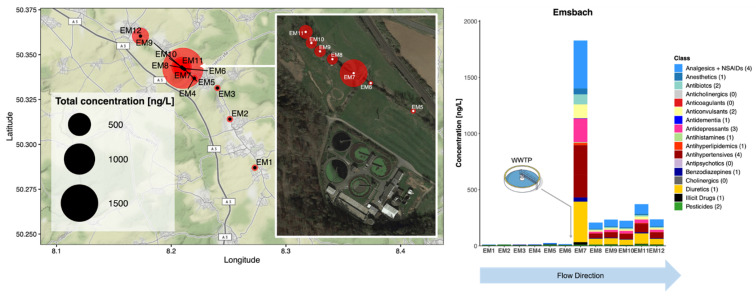
Occurrence of all CECs monitored in the River Emsbach (SZ) in the Limburg-Weilburg district, Hesse, Germany, in grab samples taken in 2020. Red circles represent sampling points (labelled EM1-12) and the size of the circle represents the combined total CEC concentration in ng/L. Individual compounds within each class can be found in Table S8.

In this rural area 25 compounds were detected at least once but were generally present at lower concentrations (Figure 3 and Table S5). Like in Campaign 1, a WWTP outfall was clearly identifiable between EM6 and EM7, where the total analyte concentration increased to ~1800 ng/L. Most CECs were absent above the WWTP. Occurrence there mostly comprised of fenuron (9 ± 2 ng/L on average across EM1-6). At EM7, 66% of the combined concentration was accounted for by hydrochlorothiazide, diclofenac, valsartan (a blood pressure medication), metoprolol, and venlafaxine (an antidepressant), in decreasing order.

**Figure 4:**
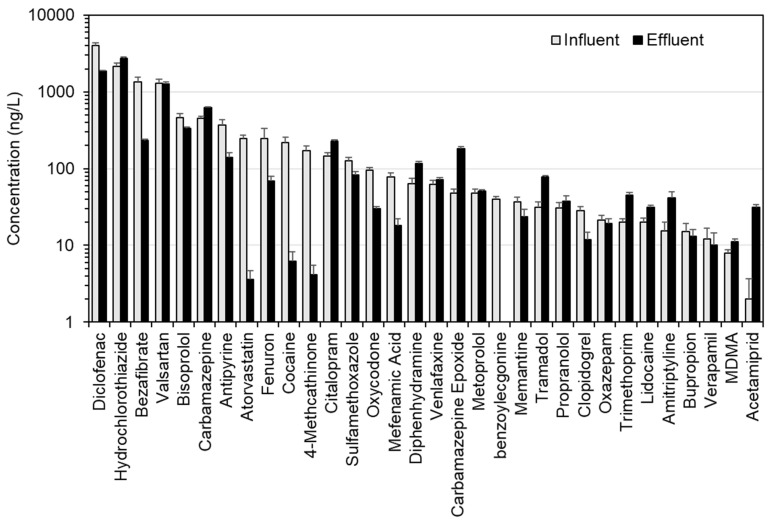
CEC concentrations in untreated and treated wastewater grab samples collected during Campaign 2 in the Selters district, Germany. Error bars represent the standard deviation of triplicate measurements.

The speed and sensitivity of the direct-injection LC-MS/MS method enabled a better understanding of the dilution of this relatively large number of CECs in this small river and at high spatial resolution. The total CEC concentration decreased by 7-fold even over a short 40 m distance, and remained consistent at this concentration for a further 3.6 km. Rúa-Gómez et al. showed that the concentrations of 3 common pharmaceuticals (tramadol, venlafaxine and lidocaine) decreased by roughly the same degree even within 60 m downstream from a WWTP outfall on the River Nidda, also located in Hesse [48]. The fenuron concentration was relatively consistent across all sites, with little discernible impact on its riverine concentration at the WWTP effluent mixing point. Analysis of the grab samples of wastewater allowed concentrations to be determined for 32 CECs including all of those measured in the river (Figure 4 and Table S7). Fenuron was determined at a concentration of 246 ± 86 ng/L (influent) and 69 ± 10 ng/L (treated wastewater), potentially explaining why its concentration did not drop in the river at the WWTP outfall. In treated wastewater, the highest measured concentrations in decreasing order were hydrochlorothiazide, diclofenac, valsartan, bisoprolol, and carbamazepine (making up >80% of total CEC concentration). This was therefore consistent with some of the most abundant CECs in the river, even though these grab samples cannot be considered representative of the temporal effluent CEC output of this WWTP.

#### 3.3.3. Campaign 3: R. Rhine, Birsig, Birs, and Wiese at Basel, Switzerland

**Figure 5:**
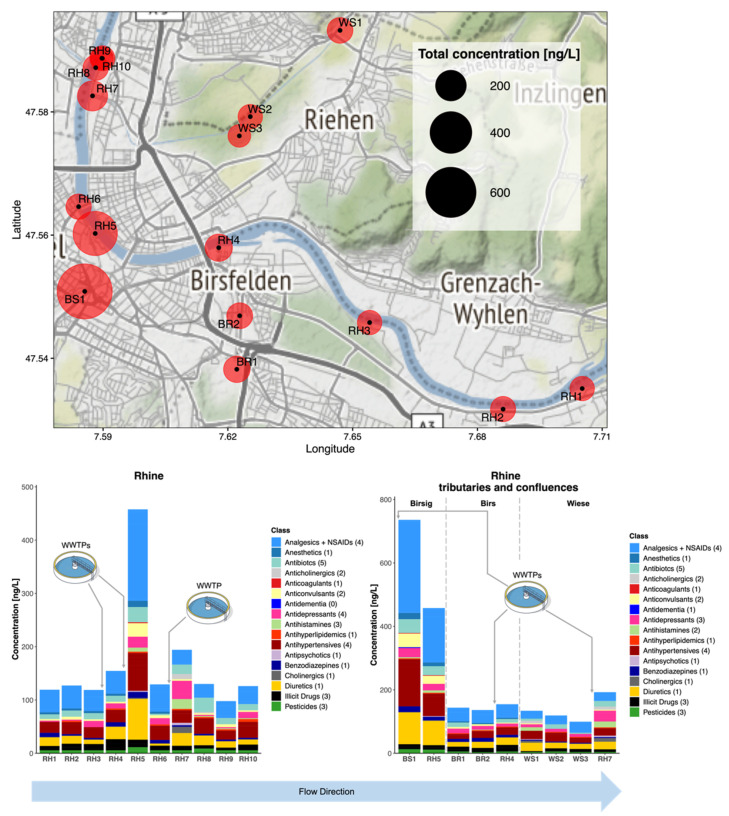
Occurrence of all CECs monitored in the River Rhine in Basel, Switzerland, along with 3 tributaries R. Birs, Birsig, and Wiese in grab samples taken in 2020/2021. Red circles represent sampling points (labelled RH1-10, BS1-2, BR1-2, and WS1-3) and the size of the circle represents the combined total CEC concentration in ng/L. Individual compounds within each class can be found in Table S8.

Despite its urban setting, CEC concentrations were the lowest in the Basel region (Figure 5 and Table S6). In 1950, the International Commission for the Protection of the Rhine (ICPR) was founded to assess and resolve pollution of the Rhine [49]. Now the Rhine is reported to be one of Europe’s cleanest rivers, having won the European River Prize awarded by the International River Foundation in 2013 [50]. In total, 40 CECs were detected across all sites in Campaign 3, with the highest combined concentration in the R. Birsig tributary at 458 ng/L. Again, significant dilution occurred over short distances from tributary confluences and WWTP sources. Across the 10 Rhine samples, the highest combined concentration of CECs was found at Schifflände (RH 5), where the R. Birsig joins the Rhine. Dominant CECs were diclofenac, hydrochlorothiazide, and valsartan (140 ± 14, 76 ± 45, and 48 ± 20 ng/L, respectively). From 2015 to 2019, these contaminants were also reported by the ICPR. In previous years, this station recorded ranges for annual average concentrations of 24.9–37.4 ng/L for diclofenac, 17.7–23.5 ng/L for hydrochlorothiazide, and 37.6–47.0 ng/L for valsartan [51]. Clearly the location of sampling for these monitoring stations is critical and this is where such a rapid method offers the most benefit for identifying localised risks. Reported ICPR concentrations coincided well with our measurements at the closest sampling sites at RH9 and 10 (diclofenac: 18.8 and 19.7 ng/L; hydrochlorothiazide: 11.9 and 9.5 ng/L; and valsartan: 10.6 and 22.4 ng/L, respectively) [51]. Only two antidepressants were found (venlafaxine and citalopram). Venlafaxine corresponded well to previous reports at 9.5 ng/L (10.73–13.20 ng/L reported from 2015 to 2019 by the ICPR) [51]. However, citalopram concentrations have not previously been reported by the ICPR in Basel and were measured here at <17 ng/L at all sites. Regarding antibiotics, only chloramphenicol, sulfamethoxazole, sulfapyridine, sulfamethazine, and trimethoprim were detected in all samples. Again, the tributaries were generally the largest contributors, with 24.3 ng/L (chloramphenicol at BS1).

### 3.4. Environmental Risk Assessment at High Spatial Resolution

**Figure 6:**
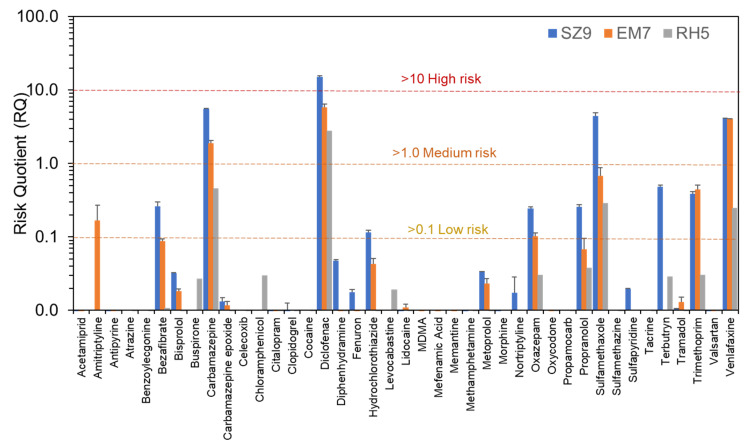
Risk quotients (MEC/PNEC) for sites with high measured CEC concentrations in the R. Schwarzach (at SZ9), Emsbach (EM7), and Rhine (RH5). Error bars represent standard deviation in RQ arising from MEC for *n* = 3. For PNEC data, please refer to Table S9.

Although run on separate days in practice, the total combined sample preparation and LC-MS/MS analysis time for all campaigns including all river and wastewater samples, blanks, matrix-matched calibrants, and quality controls was only ~53 h. This method therefore represented an excellent means to practically and rapidly assess CEC risks at scale. Based on all CECs concentrations determined, RQs for 41 compounds were calculated (Figure 6 and Table S9). Of these, four compounds had a RQ > 1, indicating medium to high risk (carbamazepine, diclofenac, sulfamethoxazole, and venlafaxine). Despite having some of the highest measured concentrations, risks from hydrochlorothiazide were either low or insignificant. Of all compounds, only diclofenac resulted in a RQ >10 in the R. Schwarzach at the WWTP outfall (RQ = 15.2). At the next sampling site downriver (SZ10, ~200 m from the outfall), the RQ reduced to 5, but still represented a medium environmental risk. Six compounds were classified at SZ9 in the low-risk category (bezafibrate, hydrochlorothiazide, oxazepam, propranolol, terbutryn, and trimethoprim). In general, RQs at this site were higher than in the other campaign locations. An upgrade of this WWTP is currently already underway.

In Campaign 2, RQs for diclofenac, carbamazepine, and venlafaxine were again among the highest (RQ > 1) at the WWTP outfall and all three presented similar medium risks (RQ = 4–6) to the aquatic environment. Their concentrations in treated wastewater samples were consistent with or lower than those found in previous studies in Germany (1867 ± 45, 621 ± 19, and 72 ± 4 ng/L, respectively) [52,53,54,55,56,57,58], but concentrations in receiving water at EM7 indicated insufficient dilution at the outfall to lower risks to an acceptable and insignificant level (293 ± 30, 94 ± 9, and 153 ± 1 ng/L) [48]. At river sampling sites further downstream, RQs decreased rapidly for both compounds and then remained consistent at 0.7–1.2 for diclofenac, 0.3–0.4 for carbamazepine, and 0.3–0.7 for venlafaxine, with no obvious further downward trends at a distance over 3.6 km. Therefore, all these compounds still represented a low to medium risk across this stretch of river, even after dilution.

Campaign 3 in the Basel area had the lowest CEC risks overall. Despite having five WWTPs located in the sampled area, only one site was identified as having noticeably higher CEC risks (RH5), where only diclofenac and carbamazepine had an RQ > 1 (1.2 and 2.8, respectively). This site was the confluence point with the R. Birsig, a short 21 km river which flows underground through the southern bank of the centre of Basel itself. It was originally used as an open sewer to remove human excreta to the R. Rhine before it was built over, and it is now enclosed within a stone tunnel. Therefore, it represents a very small flow and volume of water, and the sample was taken directly at the mixing point of the outflow and the R. Rhine itself. Birsig WWTP discharges treated effluent ~7 km upstream and were likely the main source of CEC contamination. Downstream in the R. Rhine, only the R. Wiese confluence resulted in any elevated CEC concentration and the associated risk was low or insignificant for all CECs (only carbamazepine, diclofenac, and venlafaxine had RQs > 0.1, with a maximum of 0.30 for diclofenac).

## 4. Conclusions

The suitability of a direct-injection LC-MS/MS method for rapid CEC monitoring and ERA was successfully demonstrated. In particular, the use of short biphenyl LC phases enabled the rapid selective separation and sensitive detection of 151 CECs, of which 102 were considered quantifiable at low ng/L concentrations. Whilst apparently matrix-dependent, filtration of samples using Teflon membranes should ideally not be performed at sites before any internal standards are added to ensure that sorption losses are accounted for before quantification is performed. The preparation and analysis of 42 river water and wastewater samples across 3 sites in Germany and Switzerland (including 6 rivers and influent/effluent at 1 WWTP), all performed in triplicate and with N ≥ 10-point matched and bracketed calibration curves, blanks, and quality controls, were possible in <3 days. Smaller wastewater-impacted sites displayed higher CEC concentrations. Samples taken from the outfall of a WWTP on the R. Schwarzach in Bavaria, Germany, contained the highest combined concentrations of 23 CECs at ~4000 ng/L, with high RQs > 10 calculated for diclofenac. In all three campaigns, CEC concentrations decreased significantly over very short distances after WWTP outfalls. Analysis of the R. Rhine in the urban Basel city area yielded low CEC concentrations overall and measurements for selected CECs were consistent with available monitoring station data in the area. Overall, this rapid analytical method provides a new solution for the rapid identification of high-risk sources of CECs and potentially at scale.

## Figures and Tables

**Table 1:** Analytical performance data for the direct-injection LC-MS/MS method performed in river water matrix according to ICH guidelines [33].

Analyte	Linearity ^a^	Peak Area Imprecision (RSD%), *n* = 6 ^b^		Matrix Effect (%), *n* = 6 ^c^		LLOD ^d^	LLOQ ^d^
	R2 (N > 5)	250 ng/L	2500 ng/L	250 ng/L	2500 ng/L	ng/L	ng/L
Pharmaceuticals (*n* = 66)							
mean	0.9930	12	5	+42	+17	4	11
standard deviation	±0.0115	±18	±3	±182	±58	±7	±22
median	0.9967	6	4	+5	+5	2	5
Illicit Drugs (*n* = 10)							
mean	0.9959	3	3	+2	−2	2	6
standard deviation	0.0050	±1	±1	±15	±13	±1	±2
median	0.9981	3	3	−1	−2	2	5
Pesticides (*n* = 37)							
mean	0.9943	7	4	−1	+1	2	5
standard deviation	0.0083	±6	±2	±10	±8	±1	±3
median	0.9982	5	4	+1	+2	1	4

^a^ Performed in background-corrected pooled river water matrix. Please refer to the Table S3 for the specific range tested for individual compounds (for 88 compounds the range was 10–10,000 ng/L); ^b^ Measured using peak area; ^c^ Positive values indicate signal enhancement and vice versa; ^d^ Calculated using the standard error in the intercept of the background-corrected matrix-matched calibration line.

## Supplementary Materials

The following are available online. S1. Reference materials lists, Figure S1: batch stability of retention time and peak area; Table S1: detailed LC-MS parameters; Table S2: analyte recovery data following filtration; Table S3: method performance data; Tables S4–S6: CEC occurrence data in Campaigns 1–3; Table S7: wastewater analysis CEC data; Table S8: compound classifications; Table S9: environmental risk assessment data.

## References

[1] Wang Z., Walker G.W., Muir D.C.G., Nagatani-Yoshida K. (2020). Toward a Global Understanding of Chemical Pollution: A First Comprehensive Analysis of National and Regional Chemical Inventories. Environ. Sci. Technol..

[2] Steffen W., Richardson K., Rockström J., Cornell S.E., Fetzer I., Bennett E.M., Biggs R., Carpenter S.R., de Vries W., de Wit C.A. (2015). Planetary boundaries: Guiding human development on a changing planet. Science.

[3] United Nations Environment Programme (2021). Making Peace with Nature: A Scientific Blueprint to Tackle the Climate, Biodiversity and Pollution Emergencies.

[4] Galani A., Alygizakis N., Aalizadeh R., Kastritis E., Dimopoulos M.-A., Thomaidis N.S. (2021). Patterns of pharmaceuticals use during the first wave of COVID-19 pandemic in Athens, Greece as revealed by wastewater-based epidemiology. Sci. Total Environ..

[5] Rodriguez-Mozaz S., Lopez de Alda M.J., Barceló D. (2007). Advantages and limitations of on-line solid phase extraction coupled to liquid chromatography–mass spectrometry technologies versus biosensors for monitoring of emerging contaminants in water. J. Chromatogr. A.

[6] Ulrich J.C., Ferguson P.L. (2021). Development of a sensitive direct injection LC-MS/MS method for the detection of glyphosate and aminomethylphosphonic acid (AMPA) in hard waters. Anal. Bioanal. Chem..

[7] Richardson A.K., Chadha M., Rapp-Wright H., Mills G.A., Fones G.R., Gravell A., Stürzenbaum S., Cowan D.A., Neep D.J., Barron L.P. (2021). Rapid direct analysis of river water and machine learning assisted suspect screening of emerging contaminants in passive sampler extracts. Anal. Methods.

[8] Ng K.T., Rapp-Wright H., Egli M., Hartmann A., Steele J.C., Sosa-Hernández J.E., Melchor-Martínez E.M., Jacobs M., White B., Regan F. (2020). High-throughput multi-residue quantification of contaminants of emerging concern in wastewaters enabled using direct injection liquid chromatography-tandem mass spectrometry. J. Hazard. Mater..

[9] Askeland M., Clarke B., Paz-Ferreiro J. (2020). A serial PFASs sorption technique coupled with adapted high volume direct aqueous injection LCMS method. MethodsX.

[10] Mosekiemang T.T., Stander M.A., de Villiers A. (2019). Simultaneous quantification of commonly prescribed antiretroviral drugs and their selected metabolites in aqueous environmental samples by direct injection and solid phase extraction liquid chromatography-tandem mass spectrometry. Chemosphere.

[11] Botero-Coy A.M., Martínez-Pachón D., Boix C., Rincón R.J., Castillo N., Arias-Marín L.P., Manrique-Losada L., Torres-Palma R., Moncayo-Lasso A., Hernández F. (2018). An investigation into the occurrence and removal of pharmaceuticals in Colombian wastewater. Sci. Total Environ..

[12] Campos-Mañas M.C., Plaza-Bolaños P., Sánchez-Pérez J.A., Malato S., Agüera A. (2017). Fast determination of pesticides and other contaminants of emerging concern in treated wastewater using direct injection coupled to highly sensitive ultra-high performance liquid chromatography-tandem mass spectrometry. J. Chromatogr. A.

[13] Pérez-Parada A., Gómez-Ramos Mdel M., Martínez Bueno M.J., Uclés S., Uclés A., Fernández-Alba A.R. (2012). Analytical improvements of hybrid LC-MS/MS techniques for the efficient evaluation of emerging contaminants in river waters: A case study of the Henares River (Madrid, Spain). Environ. Sci. Pollut. Res. Int..

[14] Busetti F., Backe W.J., Bendixen N., Maier U., Place B., Giger W., Field J.A. (2012). Trace analysis of environmental matrices by large-volume injection and liquid chromatography-mass spectrometry. Anal. Bioanal. Chem..

[15] Furlong E.T., Noriega M.C., Kanagy C.J., Kanagy L.K., Coffey L.J., Burkhardt M.R. (2014). Determination of Human-Use Pharmaceuticals in Filtered Water by Direct Aqueous Injection: High-Performance Liquid Chromatography/Tandem Mass Spectrometry.

[16] Oliveira T.S., Murphy M., Mendola N., Wong V., Carlson D., Waring L. (2015). Characterization of Pharmaceuticals and Personal Care products in hospital effluent and waste water influent/effluent by direct-injection LC-MS-MS. Sci. Total Environ..

[17] Hermes N., Jewell K.S., Wick A., Ternes T.A. (2018). Quantification of more than 150 micropollutants including transformation products in aqueous samples by liquid chromatography-tandem mass spectrometry using scheduled multiple reaction monitoring. J. Chromatogr. A.

[18] Martínez Bueno M.J., Uclés S., Hernando M.D., Fernández-Alba A.R. (2011). Development of a solvent-free method for the simultaneous identification/quantification of drugs of abuse and their metabolites in environmental water by LC–MS/MS. Talanta.

[19] Albergamo V., Helmus R., de Voogt P. (2018). Direct injection analysis of polar micropollutants in natural drinking water sources with biphenyl liquid chromatography coupled to high-resolution time-of-flight mass spectrometry. J. Chromatogr. A.

[20] Couchman L., Fisher D.S., Subramaniam K., Handley S.A., Boughtflower R.J., Benton C.M., Flanagan R.J. (2018). Ultra-fast LC–MS/MS in therapeutic drug monitoring: Quantification of clozapine and norclozapine in human plasma. Drug Test. Anal..

[21] Baker D.R., Kasprzyk-Hordern B. (2011). Critical evaluation of methodology commonly used in sample collection, storage and preparation for the analysis of pharmaceuticals and illicit drugs in surface water and wastewater by solid phase extraction and liquid chromatography–mass spectrometry. J. Chromatogr. A.

[22] Lin X., Choi P.M., Thompson J., Reeks T., Verhagen R., Tscharke B.J., O’Malley E., Shimko K.M., Guo X., Thomas K.V. (2021). Systematic Evaluation of the In-Sample Stability of Selected Pharmaceuticals, Illicit Drugs, and Their Metabolites in Wastewater. Environ. Sci. Technol..

[23] Daten & Fakten: Unser Haus in Zahlen. https://www.sana.de/rummelsberg/ueber-uns/zahlen-fakten.

[24] Anlagen/Standorte. https://www.kbv-badcamberg.de/abwasser/anlagen--standorte.html.

[25] Gesundwerden Inmitten der Taunuslandschaft. https://www.medicalpark.de/de/Kliniken_und_Zentren/Bad_Camberg.html.

[26] MEDIAN Hohenfeld-Klinik Bad Camberg. https://www.median-kliniken.de/de/median-hohenfeld-klinik-bad-camberg/behandlungsgebiete/psychosomatik/.

[27] ARA Basel. https://www.prorheno.ch/anlagen/ara-basel.

[28] ARA Chemie. https://www.prorheno.ch/anlagen/ara-chemie.

[29] Anlagen Birs- und Birsigtal. https://www.baselland.ch/politik-und-behorden/direktionen/bau-und-umweltschutzdirektion/industrielle-betriebe/abwasseranlagen/anlagen-birs-und-birsigtal.

[30] ARA Birsig, Therwil. https://www.baselland.ch/politik-und-behorden/direktionen/bau-und-umweltschutzdirektion/industrielle-betriebe/abwasseranlagen/anlagen-birs-und-birsigtal/downloads/ara-birsig-therwil.pdf/@@download/file/ARA%20Birsig%20-%20Therwil.pdf.

[31] ARA Birs, Birsfelden. https://www.baselland.ch/politik-und-behorden/direktionen/bau-und-umweltschutzdirektion/industrielle-betriebe/abwasseranlagen/anlagen-birs-und-birsigtal/downloads/ara-birs-birsfelden.pdf/@@download/file/ARA%20Birs%20-%20Birsfelden.pdf.

[32] ARA Rhein. https://www.ararhein.ch/.

[33] ICH Harmonised Tripartite Guideline (2005). Validation of Analytical Procedures: Text and Methodology. Q2 (R1).

[34] aus der Beek T., Weber F.-A., Bergmann A., Hickmann S., Ebert I., Hein A., Küster A. (2016). Pharmaceuticals in the environment—Global occurrences and perspectives. Environ. Toxicol. Chem..

[35] Seitz W., Winzenbacher R. (2017). A survey on trace organic chemicals in a German water protection area and the proposal of relevant indicators for anthropogenic influences. Environ. Monit. Assess..

[36] (2002). Commission Decision 2002/657/EC of 12 August implementing Council Directive 96/23/EC concerning performance of analytical methods and the interpretation of results. Off. J. Eur. Communities.

[37] (2003). 37. Technical Guidance Document on Risk Assessment in Support of Commission Directive 93/67/ EEC on Risk Assessment for New Notified Substances and Commission Regulation (EC) No. 1488/94 on Risk Assessment for Existing Substances. Part II. Eur. Chem. Bur..

[38] Palma P., Köck-Schulmeyer M., Alvarenga P., Ledo L., Barbosa I.R., López de Alda M., Barceló D. (2014). Risk assessment of pesticides detected in surface water of the Alqueva reservoir (Guadiana basin, southern of Portugal). Sci. Total Environ..

[39] Vestel J., Caldwell D.J., Constantine L., D’Aco V.J., Davidson T., Dolan D.G., Millard S.P., Murray-Smith R., Parke N.J., Ryan J.J. (2016). Use of acute and chronic ecotoxicity data in environmental risk assessment of pharmaceuticals. Environ. Toxicol. Chem..

[40] Aalizadeh R., von der Ohe P.C., Thomaidis N.S. (2017). Prediction of acute toxicity of emerging contaminants on the water flea Daphnia magna by Ant Colony Optimization–Support Vector Machine QSTR models. Environ. Sci. Process. Impacts.

[41] NORMAN Ecotoxicology Database—Lowest PNECs (Verified). https://www.norman-network.com/nds/ecotox/lowestPnecsIndex.php.

[42] Borrull J., Colom A., Fabregas J., Pocurull E., Borrull F. (2019). A simple, fast method for the analysis of 20 contaminants of emerging concern in river water using large-volume direct injection liquid chromatography-tandem mass spectrometry. Anal. Bioanal. Chem..

[43] Wilkinson J.L., Boxall A.B.A., Kolpin D.W. (2019). A Novel Method to Characterise Levels of Pharmaceutical Pollution in Large-Scale Aquatic Monitoring Campaigns. Appl. Sci..

[44] Navarro S., Hernández-Bastida J., Cazaña G., Pérez-Lucas G., Fenoll J. (2012). Assessment of the Leaching Potential of 12 Substituted Phenylurea Herbicides in Two Agricultural Soils under Laboratory Conditions. J. Agric. Food Chem..

[45] Miller T.H., Ng K.T., Lamphiere A., Cameron T.C., Bury N.R., Barron L.P. (2021). Multicompartment and cross-species monitoring of contaminants of emerging concern in an estuarine habitat. Environ. Pollut..

[46] Miller T.H., Ng K.T., Bury S.T., Bury S.E., Bury N.R., Barron L.P. (2019). Biomonitoring of pesticides, pharmaceuticals and illicit drugs in a freshwater invertebrate to estimate toxic or effect pressure. Environ. Int..

[47] Water Quality Monitoring Data GC-MS and LC-MS Semi-Quantitative Screen. https://environment.data.gov.uk/portalstg/home/item.html?id=b76f059c3f294678840a2590f61f7e59.

[48] Rúa-Gómez P.C., Püttmann W. (2012). Impact of wastewater treatment plant discharge of lidocaine, tramadol, venlafaxine and their metabolites on the quality of surface waters and groundwater. J. Environ. Monit..

[49] ICPR—International Commission for the Protection of the Rhine. https://www.iksr.org/en/.

[50] River Rhine Commended for River Basin Management. https://www.eea.europa.eu/highlights/river-rhine-commended-for-river.

[51] Long-Term Annual Average Concentrations (Weil am Rhein). http://iksr.bafg.de/iksr/lj_auswahl.asp?S=3.

[52] Stülten D., Zühlke S., Lamshöft M., Spiteller M. (2008). Occurrence of diclofenac and selected metabolites in sewage effluents. Sci. Total Environ..

[53] Letzel M., Metzner G., Letzel T. (2009). Exposure assessment of the pharmaceutical diclofenac based on long-term measurements of the aquatic input. Environ. Int..

[54] Heberer T. (2002). Occurrence, fate, and removal of pharmaceutical residues in the aquatic environment: A review of recent research data. Toxicol. Lett..

[55] Quintana J.B., Carpinteiro J., Rodríguez I., Lorenzo R.A., Carro A.M., Cela R. (2004). Determination of natural and synthetic estrogens in water by gas chromatography with mass spectrometric detection. J. Chromatogr. A.

[56] Schlüsener M.P., Hardenbicker P., Nilson E., Schulz M., Viergutz C., Ternes T.A. (2015). Occurrence of venlafaxine, other antidepressants and selected metabolites in the Rhine catchment in the face of climate change. Environ. Pollut..

[57] Schröder P., Helmreich B., Škrbić B., Carballa M., Papa M., Pastore C., Emre Z., Oehmen A., Langenhoff A., Molinos M. (2016). Status of hormones and painkillers in wastewater effluents across several European states—considerations for the EU watch list concerning estradiols and diclofenac. Environ. Sci. Pollut. Res..

[58] Meyer W., Reich M., Beier S., Behrendt J., Gulyas H., Otterpohl R. (2016). Measured and predicted environmental concentrations of carbamazepine, diclofenac, and metoprolol in small and medium rivers in northern Germany. Environ. Monit. Assess..

