# Supplementary material for: Quantitative Determination and Environmental Risk Assessment of 102 Chemicals of Emerging Concern in Wastewater-Impacted Rivers Using Rapid Direct-Injection Liquid Chromatography—Tandem Mass Spectrometry
Source: Molecules. 2021 Sep 7;26(18):5431. doi: 10.3390/molecules26185431 (PMC8466042; doi:10.3390/molecules26185431)
Supplement: Supplementary file 1 [file molecules-26-05431-s001.zip › molecules-1359634-supplementary.pdf]

SUPPLEMENTARY INFORMATION

QUANTITATIVE DETERMINATION AND ENVIRONMENTAL RISK ASSESSMENT OF 102 CHEMICALS OF EMERGING CONCERN IN WASTEWATER-IMPACTED RIVERS USING RAPID DIRECT-INJECTION LIQUID CHROMATOGRAPHY TANDEM MASS SPECTROMETRY

Melanie Egli[a,1], Alicia Hartmann[a,b,1], Helena Rapp Wright[a], Keng Tiong Ng[a], Frédéric B. Piel[a] and Leon P. Barron*[a]

[a] MRC Centre for Environment and Health, School of Public Health, Imperial College London, 86 Wood Lane, London W12 0BZ, United Kingdom

[b] Hochschule Fresenius gem. GmbH, Limburger Str. 2, Idstein, 65510, Germany

[1] these authors share first authorship

* corresponding author (e-mail address: leon.barron@imperial.ac.uk)

S1. Reference materials

Reference standards (purity > 97 %) for 2-(thiocyanomethylthio)benzothiazole, 4-fluoromethacationone, 4-methylethcathinone, acetamiprid, aclinofen, alprazolam, ametryn, amiodarone-HCl, amitriptyline, amlodipine, amphetamine, antipyrine, atorvastatin, atrazine, azelnidipine, azithromycin, azoxystrobin, benoxacor, bensulide, benzatropine, benzoylecgonine (BZE), betaxolol, bezafibrate, bisoprolol, bupropion, buspirone-HCl, carazolol, carbamazepine, carboxine, carfentrazone-ethyl, carbamazepine-10,11-epoxide, celecoxib, chloramphenicol, cilazapril, citalopram-HBr, clarithromycin, clodinafop-propargyl, clofibric acid, clopidogrel-HSO_4^-, clothianidin, clozapine, cocaine, cyclouron, cycloxyidim, cymoxanil, cyphenothrin, cyromazine, diazepam, diclofenac, diflubenzuron, dimethametryn, dimethomorph, diuron, diphenhydramine-HCl, disulfoton sulfone, enalapril, ethofumesate, famoxadone, fenoxaprop-p-ethyl, fenuron, flufenoxuron, fluocinonide, fluoxetine, flurbiprophen, flurochloridone, flutamide, flutolanil, fuberidazole, gemfibrozil, haloperidol, hydrochlorothiazide, ibuprofen, imidacloprid, indomethacin, isocarbamid, isradipine, josamycin, ketamine, ketoconazole, ketoprofen, ketotifen, levamisole-HCl, levocabastine-HCl, lidocaine, lincomycin, lorazepam, 3,4-methylenedioxymethamphetamine (MDMA), meclizine-HCl, meclofenamic acid, medroxyprogesterone, mefenamic acid, memantine-HCl, mephedrone, mephosfolan, metformin, methamphetamine, methcathinone, methedrone, methylphenidate-HCl, metoprolol, morphine, nadolol, naproxen, nicotine, nifedipine, nitenpyram, nordiazepam, norethisterone, nortriptyline, orphenadrine, oxamyl, oxazepam, oxycarboxin, oxycodone-HCl, picoxystrobin, piperophos, pirenzepine-2HCl, pretilachlor, prodiamine, prometon, prometryn, propamocarb, propranolol, propazine, pymetrozine, pyracarbolid, pyraclostrobin, pyraflufen-ethyl, pyridaben, risperidone, rizatriptan, ronidazole, roxithromycin, salbumatol, salicylic acid, sertraline, simazine, spinosyn A, spinosyn D, spiramycin, sulfadimethoxine, sulfamerazine, sulfamethazine, sulfamethoxazole, sulfamonomethoxine, sulfapyridine, sulfathiazole, sulfisoxazole, tacrine, tamsulosin-HCl, temazepam, terbutryn, terfenadine, thiacloprid, thiamethoxam, thiazopyr, timolol, tramadol-HCl, trimethoprim, valsartan, venlafaxine-HCl, verapamil-HCl, warfarin and ziprasidone-HCl were acquired from Sigma-Aldrich (Steinheim, Germany). Trimethoprim was obtained from Fluka (Buchs, Switzerland). Stable isotope-labelled standards including amitriptyline-d3-HCl, amphetamine-d6, BZE-d3, cetirizine-d4, clothianidin-d3, cocaine-d3, cotinine-d3, diazepam-d6, fluoxetine-d6, gemfibrozil-d6, haloperidol-d4, ketamine-d4-HCl, lorazepam-d4, MDMA-d5, methylone-d3, methylphenidate-d9, morphine-d3, nicotine-d4, nifedipine-d4, nordiazepam-d5, nortriptyline-d3-HCl, oxazepam-d5, risperidone-d4, sertraline-d3-HCl, temazepam-d5, thiamethoxam-d3, tramadol-13C1-d3-HCl, venlafaxine-d6-HCl, were purchased from Sigma Aldrich. Betaxolol-d7-HCl, celecoxib-d7, clarithromycin-d3, lidocaine-d10-HCl, metoprolol-d7-HCl, sulfamethazine-d4, trimethoprim-d3 and verapamil-d3-HCl were ordered from QMX (Essex, UK).

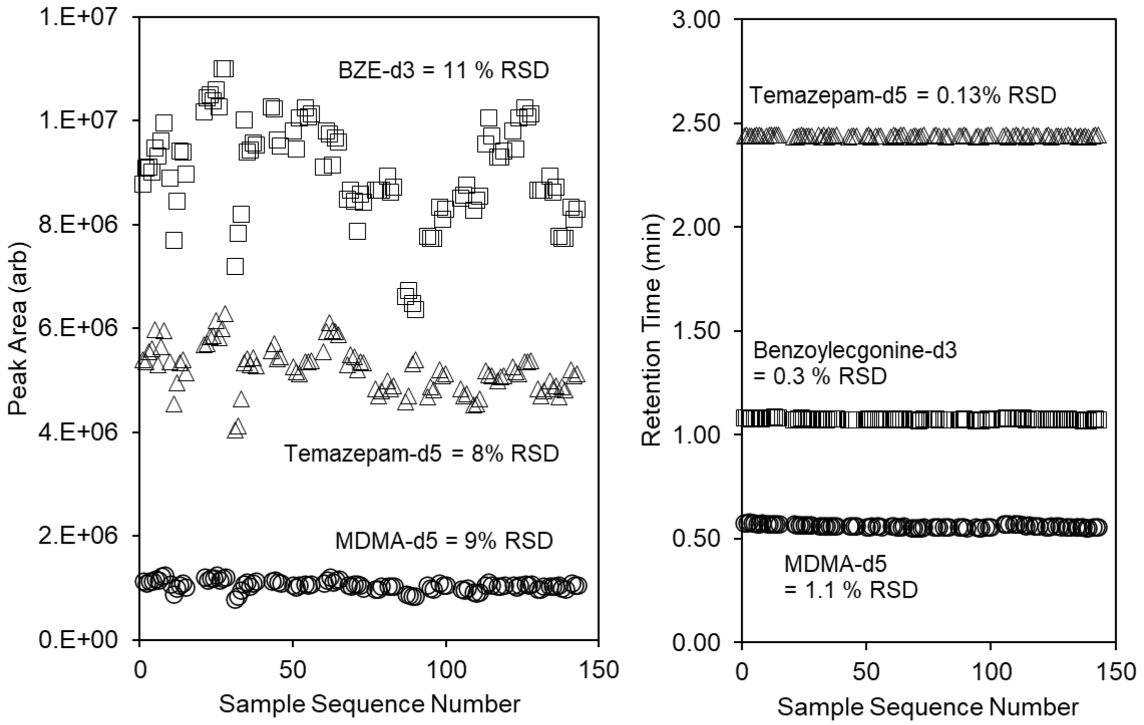

Figure S1. Examples of stability of peak area (left) and retention time (right) for three selected SIL-IS over a 144-sample injection sequence.

Table S1. MRM transitions and other MS parameters for all CECs and SIL-IS.

Compounds	Precursor (m/z)	Transition (m/z)	Polarity	Pause Time (ms)	Dwell Time (ms)	Q1 Pre Bias (V)	Collision Energy (V)	Q3 Pre Bias (V)	Retention Time Window Range (min)	
2-(Thiocyanomethylthio) benzothiazole	238.7	180.1	+	2	2	-27	-14	-19	2.34	3.14
		136.1	+	2	2	-29	-26	-14		
4-Fluoromethacationone	182.1	164.2	+	2	5	-30	-15	-17	0.00	0.71
		149.1	+	2	5	-13	-22	-27		
4-Methylethcathinone	192.1	174.2	+	2	5	-23	-16	-18	0.40	1.20
		144.1	+	2	5	-10	-29	-25		
Acetamiprid	233	126.1	+	2	13	-16	-21	-13	1.12	1.92
Alprazolam	309.1	281.1	+	2	2	-16	-26	-22	2.04	2.84
		205.1	+	2	2	-16	-40	-23		
Ametryn	228.1	186.1	+	2	4	-11	-20	-20	1.17	1.97
		96.1	+	2	4	-23	-28	-19		
Amiodarone	645.8	58.2	+	2	20	-32	-49	-27	3.07	3.67
		100.2	+	2	20	-32	-30	-10		
Amitriptyline	278.2	91.1	+	2	4	-13	-27	-19	1.87	2.67
		105.1	+	2	4	-13	-25	-21		
Amitriptyline-d3	281.1	233.2	+	2	4	-19	-17	-25	1.86	2.66
		105.2	+	2	4	-10	-23	-19		
Amlodipine	409.1	238.2	+	2	6	-15	-12	-24	1.84	2.64
Amphetamine	136.1	91.1	+	2	3	-14	-17	-19	0.00	0.61
		119.1	+	2	3	-14	-15	-25		
Amphetamine-d6	142.2	93.2	+	2	3	-17	-19	-17	0.00	0.67
		125.2	+	2	3	-16	-14	-21		
Antipyrine	188.9	77.2	+	2	5	-13	-40	-30	0.73	1.53
		56.2	+	2	5	-13	-32	-23		
Atorvastatin	559.1	440.3	+	2	5	-20	-23	-16	2.78	3.58
		250.2	+	2	5	-20	-43	-27		
Atrazine	216.1	174.1	+	2	14	-11	-18	-18	1.38	2.18
Azelnidipine	583.2	167.2	+	2	2	-40	-27	-30	2.70	3.50
		165.2	+	2	2	-40	-55	-16		
Azithromycin	749.6	591.4	+	2	2	-28	-30	-22	1.08	1.88
		158.3	+	2	2	-28	-40	-16		
Azoxystrobin	404.2	372.1	+	2	2	-21	-16	-27	2.63	3.43
		344.1	+	2	2	-21	-26	-24		
Benoxacor	260.0	149.2	+	2	2	-30	-18	-27	2.10	2.90
		134.1	+	2	2	-30	-29	-25		
Bensulide	398.1	158.1	+	2	1	-20	-24	-16	2.90	3.70
		217.9	+	2	1	-20	-17	-23		
Benzatropine	308.1	167.2	+	2	5	-11	-30	-17	1.91	2.71
		165.2	+	2	5	-21	-52	-17		
Benzoylecgonine	290.1	168.2	+	2	11	-20	-19	-18	0.67	1.47
Benzoylecgonine-d3	293.1	171.2	+	2	11	-30	-20	-18	0.67	1.47
Betaxolol	308.3	116.2	+	2	4	-15	-21	-21	1.28	2.08
		72.2	+	2	4	-21	-24	-27		
Bezafibrate	360.2	274.0	-	2	5	18	17	12	2.17	2.97
		154.1	-	2	5	20	29	15		
Bisoprolol	326.2	116.2	+	2	5	-23	-19	-21	1.05	1.85
		74.2	+	2	5	-12	-26	-29		
Bupropion	240.1	184.2	+	2	4	-29	-13	-12	0.88	1.68
		131.2	+	2	4	-27	-26	-13		
Buspirone	386.1	122.2	+	2	2	-26	-30	-23	1.39	2.19
		109.2	+	2	2	-25	-46	-11		
Carazolol	299.1	116.2	+	2	6	-21	-21	-20	1.22	2.02
		222.2	+	2	6	-21	-21	-24		
Carbamazepine	237.1	194.0	+	2	4	-12	-20	-23	1.61	2.41
		192.1	+	2	4	-12	-25	-22		
Carbamazepine epoxide	252.9	180.2	+	2	4	-17	-29	-18	1.30	2.10
		236.2	+	2	4	-17	-12	-16		
Carboxine	236.0	143.1	+	2	13	-26	-15	-14	1.67	2.47
Carfentrazone-ethyl	412.1	346.0	+	2	2	-19	-17	-24	2.71	3.51
		366.0	+	2	2	-19	-19	-21		
Celecoxib	382.1	362.1	+	2	4	-11	-28	-25	2.63	3.43
		300.2	+	2	4	-18	-28	-21		

Celecoxib-d7	389.0	369.1	+	2	2	-19	-29	-25	2.62	3.42
		289.2	+	2	2	-26	-37	-30		
Chloramphenicol	322.7	152.2	-	2	5	16	17	15	0.98	1.78
		257.0	-	2	5	17	11	25		
Cilazapril	418.0	211.2	+	2	5	-20	-20	-20	1.73	2.53
		70.2	+	2	5	-15	-46	-12		
Citalopram	325.1	109.2	+	2	4	-22	-26	-20	1.53	2.33
		262.2	+	2	4	-24	-20	-30		
Clarithromycin	748.2	158.2	+	2	2	-36	-34	-10	1.85	2.65
		290.4	+	2	2	-36	-20	-22		
Clarithromycin-d3	751.2	161.2	+	2	2	-38	-28	-16	1.85	2.65
		593.4	+	2	2	-38	-22	-22		
Clodinafop-propargyl	350.1	266.1	+	2	1	-18	-17	-19	2.93	3.73
		238.2	+	2	1	-18	-27	-24		
Clofibric acid	212.5	126.9	-	2	11	11	15	24	2.07	2.87
Clopidogrel	322.0	212.2	+	2	2	-23	-17	-22	1.95	2.75
		184.1	+	2	2	-22	-22	-19		
Clothianidin	250.1	132.0	+	2	3	-12	-15	-13	0.61	1.41
		169.1	+	2	3	-12	-13	-18		
Clozapine	327.2	270.2	+	2	2	-16	-23	-20	1.08	1.88
		192.1	+	2	2	-16	-45	-14		
Cocaine	304.2	182.3	+	2	5	-11	-21	-14	0.99	1.79
		82.1	+	2	5	-11	-35	-17		
Cocaine-d3	307.1	185.2	+	2	5	-22	-21	-19	0.99	1.79
		77.2	+	2	5	-15	-54	-14		
Cyclouron	199.1	72.2	+	2	3	-14	-25	-13	1.54	2.34
		89.2	+	2	3	-22	-14	-16		
Cycloxyidim	326.1	280.2	+	2	5	-16	-14	-19	2.87	3.67
		180.2	+	2	5	-12	-22	-19		
Cymoxanil	199.2	111.2	+	2	3	-23	-19	-18	0.83	1.63
		83.0	+	2	3	-23	-27	-17		
Cyromazine	167.2	68.0	+	2	9	-19	-32	-26	0.00	0.45
		59.9	+	2	9	-19	-22	-24		
Diazepam	285.1	154.1	+	2	2	-11	-28	-18	2.19	2.99
		193.1	+	2	2	-11	-29	-23		
Diazepam-d6	289.9	198.2	+	2	2	-19	-33	-21	2.17	2.97
		154.2	+	2	2	-19	-27	-15		
Diclofenac	296.0	215.1	+	2	6	-15	-20	-16	2.58	3.38
		214.0	+	2	6	-15	-40	-24		
Diflubenzuron	311.0	158.1	+	2	1	-16	-16	-29	2.50	3.30
		141.1	+	2	1	-16	-32	-24		
Dimethametryn	256.1	186.2	+	2	2	-28	-22	-30	1.69	2.49
		68.1	+	2	2	-13	-44	-11		
Dimethomorph	388.2	301.1	+	2	2	-20	-21	-21	2.41	3.21
		165.1	+	2	2	-20	-35	-17		
Diphenhydramine	256	167.1	+	2	2	-30	-11	-12	1.42	2.22
		152.0	+	2	2	-30	-40	-17		
Enalapril	375.1	114.0	-	2	15	27	24	21	1.34	2.14
Famoxadone	375.8	196.1	+	2	6	-25	-21	-20	2.97	3.77
Fenoxaprop-ethyl	362.1	288.1	+	2	2	-18	-18	-21	3.00	3.80
		121.1	+	2	2	-19	-33	-13		
Fenuron	165.0	72.2	+	2	5	-18	-22	-28	0.40	1.20
		46.1	+	2	5	-18	-14	-18		
Flufenoxuron	489.1	158.1	+	2	2	-15	-21	-17	3.04	3.84
		141.0	+	2	2	-24	-39	-15		
Fluocinonide	494.9	337.2	+	2	6	-23	-19	-25	2.59	3.39
Fluoxetine	310.2	44.2	+	2	2	-15	-16	-17	1.79	2.59
		148.2	+	2	2	-15	-10	-11		
Fluoxetine-d6	316.1	44.2	+	2	2	-20	-15	-20	1.78	2.58
		154.3	+	2	2	-15	-9	-16		
Flurbiprofen	245.2	188.7	+	2	6	-24	-15	-29	1.46	2.26
Flurochloridone	312.0	291.9	+	2	2	-16	-21	-20	2.52	3.32
		144.9	+	2	2	-16	-46	-29		
Flutamide	275.0	202.0	-	2	2	14	23	20	2.10	2.90
		205.0	-	2	2	13	21	23		
Flutolanil	324.3	242.2	+	2	2	-15	-26	-16	2.46	3.26
		262.2	+	2	2	-15	-19	-18		
Fuberidazole	184.9	157.2	+	2	5	-23	-21	-16	0.07	0.87
		156.2	+	2	5	-23	-27	-16		
Hydrochlorothiazide	296.0	269.0	-	2	10	11	19	11	0.04	0.84
Imidacloprid	256.1	175.1	+	2	5	-13	-19	-20	0.97	1.76

		209.1	+	2	5	-13	-18	-10		
Indomethacin	356.5	312.3	-	2	14	18	11	10	2.71	3.51
Isocarbamid	186.1	87.0	+	2	2	-20	-16	-15	0.53	1.33
		44.2	+	2	2	-20	-32	-17		
Isradipine	370.2	119.0	-	2	2	14	16	11	2.42	3.22
		250.0	-	2	2	11	16	17		
Josamycin	828.5	109.3	+	2	6	-30	-47	-27	2.24	3.04
Ketamine	238.1	125.0	+	2	5	-12	-26	-26	0.65	1.45
		207.0	+	2	5	-12	-15	-15		
Ketamine-d4	242.1	129.2	+	2	5	-25	-28	-26	0.63	1.43
		211.3	+	2	5	-28	-15	-10		
Ketoconazole	533.0	491.1	+	2	4	-36	-31	-24	2.08	2.88
		82.2	+	2	4	-36	-47	-14		
Ketoprofen	253.1	209.1	-	2	3	23	8	20	2.06	2.86
		221.1	-	2	3	13	17	14		
Ketotifen	310.0	96.2	+	2	10	-22	-24	-18	1.30	2.10
Levamisole	207.1	180.0	+	2	10	-10	-24	-20	0.14	0.94
		91.1	+	2	10	-10	-41	-17		
Levocabastine	421.4	174.2	+	2	6	-20	-32	-18	1.80	2.60
		70.2	+	2	6	-15	-37	-12		
Lidocaine	235.0	86.1	+	2	5	-26	-19	-18	0.11	0.91
		58.1	+	2	5	-26	-45	-12		
Lidocaine-d10	245.2	96.3	+	2	13	-29	-22	-17	0.11	0.91
Lincomycin	407.2	126.2	+	2	5	-22	-31	-13	0.00	0.72
		359.2	+	2	5	-23	-19	-27		
Lorazepam	321.1	303.0	+	2	5	-12	-10	-24	1.82	2.62
		274.9	+	2	5	-12	-22	-22		
Lorazepam-d4	325.1	279.1	+	2	5	-22	-23	-19	1.81	2.61
		307.1	+	2	5	-23	-17	-21		
MDMA	194.1	163.1	+	2	5	-10	-13	-12	0.15	0.95
		105.1	+	2	5	-10	-25	-22		
Meclizine	391.1	201.1	+	2	1	-28	-20	-22	2.61	3.41
		165.1	+	2	1	-19	-55	-17		
Medroxyprogesterone	345.1	123.2	+	2	2	-23	-26	-21	2.61	3.41
		97.2	+	2	2	-23	-27	-10		
Mefenamic acid	240.0	196.2	-	2	12	17	18	12	2.72	3.52
Memantine	180.3	163.3	+	2	6	-19	-18	-17	0.85	1.65
		107.3	+	2	6	-20	-26	-20		
Mephedrone	178.3	145.1	+	1	10	-20	-21	-29	0.21	1.01
		160.3	+	1	10	-20	-16	-11		
Mephosfolan	270.1	140.0	+	2	12	-13	-25	-14	1.40	2.20
Metformin	130.3	71.2	+	2	10	-23	-23	-12	0.00	0.49
		60.2	+	2	10	-14	-15	-26		
Methamphetamine	150.1	91.1	+	2	5	-10	-19	-18	0.00	0.78
		119.1	+	2	5	-10	-10	-24		
Methcathinone	164.0	131.2	+	2	5	-29	-21	-25	0.00	0.67
		146.2	+	2	5	-11	-16	-27		
Methedrone	194.3	176.2	+	2	5	-20	-15	-29	0.12	0.92
		161.1	+	2	5	-21	-21	-16		
Methylone-d3	211.1	163.2	+	2	5	-24	-17	-28	0.00	0.80
		135.2	+	2	5	-24	-27	-13		
Methylphenidate	234.2	84.1	+	2	6	-11	-20	-17	0.77	1.57
		56.1	+	2	6	-11	-45	-22		
Methylphenidate-d9	243.3	93.3	+	5	6	-28	-24	-17	0.75	1.55
		61.2	+	2	6	-27	-50	-23		
Metoprolol	268.2	116.2	+	2	5	-30	-21	-21	0.70	1.50
		159.1	+	2	5	-10	-22	-16		
Metoprolol-d7	275.2	123.2	+	2	5	-22	-21	-23	0.69	1.49
		105.2	+	2	5	-29	-22	-19		
Morphine	286.2	165.1	+	2	5	-14	-44	-12	0.00	0.60
		153.1	+	2	5	-14	-45	-29		
Morphine-d3	289.0	152.2	+	2	5	-14	-55	-30	0.00	0.59
		201.2	+	2	5	-14	-27	-21		
Nadolol	310.3	254.2	+	2	3	-21	-17	-17	0.24	1.04
		236.2	+	2	3	-21	-21	-25		
Nicotine	163.0	117.2	+	2	15	-20	-30	-20	0.00	0.51
		130.2	+	2	15	-20	-25	-20		
Nicotine-d4	167.0	134.2	+	2	15	-12	-22	-25	0.00	0.55
		121.2	+	2	15	-18	-28	-21		
Nifedipine	345.2	222.1	-	2	3	10	10	15	2.12	2.92
		122.0	-	2	3	13	12	27		

Nifedipine-d4	349.0	222.1	-	2	3	10	10	24	2.10	2.90
		126.1	-	2	3	13	12	28		
Nitenpyram	27100	225.2	+	2	6	-19	-12	-15	0.11	0.91
		126.2	+	2	6	-19	-28	-13		
Nordiazepam	270.8	140.2	+	2	2	-18	-28	-14	1.76	2.56
		208.1	+	2	2	-10	-28	-14		
Norethisterone	299.1	109.2	+	2	2	-21	-26	-19	2.46	3.26
		231.2	+	2	2	-11	-20	-11		
Nortriptyline	264.2	233.1	+	2	4	-10	-15	-17	1.82	2.62
		91.1	+	2	4	-10	-25	-18		
Nortriptyline-d3	267.0	233.2	+	2	4	-18	-15	-16	1.82	2.62
		105.2	+	2	4	-18	-22	-19		
Orphenadrine	270.1	181.2	+	2	4	-29	-13	-29	1.61	2.41
		166.1	+	2	4	-10	-28	-30		
Oxamyl	237.1	72.1	+	2	4	-12	-12	-13	0.14	0.94
		90.2	+	2	4	-12	-10	-17		
Oxycarboxin	268.1	175.0	+	2	5	-13	-14	-18	1.10	1.90
		147.0	+	2	5	-13	-27	-30		
Oxycodone	316.2	298.2	+	2	20	-16	-19	-22	0.30	1.10
		241.1	+	2	20	-16	-30	-18		
Picoxystrobin	368.0	145.2	+	2	1	-25	-22	-15	2.82	3.62
		205.2	+	2	1	-25	-10	-14		
Piperophos	353.9	171.1	+	2	2	-23	-22	-18	2.97	3.77
		255.1	+	2	2	-22	-14	-12		
Pirenzipine	352.1	113.3	+	2	5	-26	-22	-22	0.40	1.20
		70.2	+	2	5	-10	-46	-12		
Pretilachlor	312.2	252.1	+	2	2	-16	-17	-17	2.87	3.67
		176.1	+	2	2	-16	-29	-18		
Prodiamine	349.2	232.0	-	2	2	10	24	10	3.00	3.80
		216.0	-	2	2	13	29	14		
Prometon	226.1	184.2	+	2	5	-11	-20	-12	0.98	1.78
		142.2	+	2	5	-11	-23	-14		
Prometryn	242.2	158.1	+	2	2	-12	-25	-16	1.42	2.22
		200.0	+	2	2	-12	-20	-22		
Propamocarb	189.2	102.1	+	2	4	-27	-17	-20	0.00	0.60
		74.2	+	2	4	-10	-26	-30		
Propanolol	260.1	116.2	+	2	4	-30	-19	-21	1.29	2.09
		183.2	+	2	4	-10	-20	-18		
Propazine	230.1	188.2	+	2	4	-26	-19	-20	1.70	2.50
		146.1	+	2	4	-25	-24	-25		
Pymetrozine	218.1	105.1	+	2	6	-11	-21	-21	0.00	0.61
		79.0	+	2	6	-11	-45	-15		
Pyracarbolid	218.1	125.1	+	2	3	-24	-18	-24	1.46	2.26
		97.1	+	2	3	-24	-28	-17		
Pyraclostrobin	390.1	194.1	+	2	2	-19	-15	-21	2.99	3.79
		163.1	+	2	2	-19	-27	-17		
Pyraflufen-ethyl	413	339.0	+	2	2	-21	-20	-23	2.87	3.67
		253.1	+	2	2	-21	-35	-26		
Risperidone	411.2	191.1	+	2	10	-12	-31	-23	1.27	2.07
		69.1	+	2	10	-12	-50	-27		
Risperidone-d4	415.2	195.2	+	2	10	-29	-30	-21	1.26	2.06
Rizatriptan	270.1	201.2	+	2	5	-10	-14	-21	0.11	0.91
		158.2	+	2	5	-20	-21	-16		
Ronidazole	201.0	140.2	+	2	11	-14	-13	-14	0.04	0.84
Roxithromycin	837.3	679.4	+	2	4	-24	-22	-24	2.00	2.80
		158.1	+	2	4	-24	-34	-16		
Salbutamol	240.1	148.2	+	2	20	-29	-20	-28	0.00	0.58
		222.3	+	2	20	-12	-12	-23		
Salicylic acid	137.2	93.1	-	2	9	20	20	16	0.57	1.37
		65.1	-	2	9	20	30	24		
Simazine	202.1	104.0	+	2	5	-23	-25	-19	1.04	1.84
		68.1	+	2	5	-23	-32	-27		
Spinosyn A	732.6	142.1	+	2	5	-22	-34	-29	2.69	3.49
		98.0	+	2	5	-22	-40	-19		
Spinosyn D	746.6	142.1	+	2	6	-22	-36	-15	2.83	3.63
		98.0	+	2	6	-22	-40	-19		
Spiramycin	843.5	174.2	+	2	10	-24	-36	-11	1.19	1.99
Sulfadimethoxine	311.1	156.0	+	1	10	-16	-20	-17	1.33	2.13
		92.1	+	1	10	-16	-32	-17		
Sulfamerazine	265.1	92.1	+	2	3	-13	-34	-17	1.82	2.62
		156.0	+	2	3	-13	-17	-16		

Sulfamethazine	278.9	186.1	+	2	6	-18	-17	-19	0.71	1.51
		124.2	+	2	6	-10	-24	-12		
Sulfamethazine-d4	282.8	186.2	+	2	3	-13	-20	-19	0.70	1.50
		124.2	+	2	3	-17	-25	-27		
Sulfamethoxazole	254.1	156.0	+	2	6	-12	-18	-16	0.94	1.74
		92.2	+	2	6	-12	-31	-16		
Sulfamonomethoxine	281.1	156.1	+	2	4	-14	-18	-17	0.84	1.64
		92.2	+	2	4	-14	-33	-18		
Sulfapyridine	250.0	156.0	+	2	4	-23	-17	-16	0.29	1.09
		92.1	+	2	4	-22	-32	-18		
Sulfathiazole	256.0	156.0	+	2	8	-24	-16	-16	0.32	1.12
		92.2	+	2	8	-24	-27	-17		
Sulfisoxazole	268.0	156.1	+	2	13	-30	-15	-16	1.04	1.84
		113.2	+	2	13	-10	-16	-11		
Tacrine	199.0	171.2	+	2	6	-23	-30	-17	0.63	1.43
		144.1	+	2	6	-14	-36	-28		
Tamsulosin	409.1	228.1	+	2	4	-28	-24	-24	1.33	2.13
		271.2	+	2	4	-28	-20	-13		
Temazepam	301.1	255.1	+	2	4	-11	-25	-30	2.07	2.87
		283.2	+	2	4	-11	-13	-23		
Temazepam-d5	306.0	260.1	+	2	10	-11	-24	-17	2.06	2.86
Terbutryn	242.1	186.1	+	2	2	-28	-20	-20	1.48	2.28
		158.2	+	2	2	-29	-24	-27		
Terfenadine	472.4	436.3	+	2	6	-24	-28	-22	2.59	3.39
		454.3	+	2	6	-13	-22	-22		
Thiacloprid	253.1	126.1	+	2	4	-13	-22	-25	1.43	2.23
		90.1	+	2	4	-13	-38	-18		
Thiamethoxam	292.0	211.1	+	2	2	-14	-13	-23	0.61	1.41
		181.0	+	2	2	-14	-24	-19		
Thiamethoxam-d3	296.6	214.1	+	2	3	-11	-12	-23	0.58	1.38
		184.1	+	2	3	-14	-24	-19		
Thiazopyr	397.0	377.1	+	2	6	-14	-23	-18	2.76	3.56
Timolol	317.1	261.1	+	2	13	-16	-17	-30	0.67	1.47
Tramadol	264.1	58.2	+	2	14	-19	-16	-22	0.72	1.52
Tramadol-13C1, d3	268.3	58.2	+	2	14	-13	-23	-24	0.71	1.51
Trimethoprim	291.1	230.1	+	2	5	-30	-25	-26	0.35	1.15
		123.2	+	2	5	-15	-28	-25		
Trimethoprim-d3	294.1	230.2	+	2	5	-20	-25	-25	0.34	1.14
		123.2	+	2	5	-11	-26	-12		
Valsartan	436.4	291.2	+	2	5	-15	-18	-14	2.40	3.20
		235.2	+	2	5	-12	-17	-16		
Venlafaxine	278.2	58.1	+	2	5	-13	-19	-22	1.03	1.83
		260.2	+	2	5	-13	-15	-20		
Venlafaxine-d6	284.2	64.2	+	2	5	-20	-23	-26	1.03	1.83
		260.2	+	2	5	-13	-15	-20		
Verapamil	455.2	165.2	+	2	2	-30	-29	-30	1.89	2.69
		414.4	+	2	2	-16	-16	-15		
Verapamil-d3	458.2	165.2	+	2	2	-30	-29	-29	1.89	2.69
		306.3	+	2	2	-30	-26	-15		
Warfarin	309.2	163.1	+	2	5	-15	-16	-19	2.26	3.06
		251.1	+	2	5	-15	-20	-29		
Ziprasidone	413.1	194.1	+	2	2	-20	-30	-20	1.65	2.45

Table S2. Recovery of CECs and SIL-IS from standard solutions and spiked freshwater river matrix at 100 ng/L for n=3 replicates each.

Compound	River water			Ultrapure water		
	Recovery ± SD (%)			Recovery ± SD (%)		
2-(Thiocyanomethylthio)benzothiazole	98	±	3	123	±	5
4-Fluoromethacationone	100	±	2	120	±	8
4-Methylethcathinone	101	±	2	115	±	7
Acetamiprid	101	±	5	112	±	3
Alprazolam	96	±	12	107	±	8
Ametryn	97	±	5	115	±	7
Amiodarone	0	±	0	0	±	0
Amitriptyline	89	±	4	99	±	3
Amitriptyline-d3	116	±	8	162	±	17
Amlodipine	77	±	14	63	±	24
Amphetamine	100	±	18	117	±	4
Amphetamine-d6	118	±	2	116	±	1
Antipyrine	97	±	5	121	±	6
Atorvastatin	67	±	6	87	±	10
Atrazine	99	±	4	113	±	2
Azelnidipine	0	±	0	0	±	0
Azithromycin	54	±	2	0	±	0
Azoxystrobin	87	±	3	97	±	4
Benoxacor	96	±	30	121	±	21
Bensulide	79	±	11	74	±	6
Benzatropine	93	±	6	100	±	4
Benzoylecgonine, BZE	100	±	9	101	±	7
Benzoylecgonine, BZE-d3	112	±	4	102	±	2
Betaxolol	99	±	19	111	±	10
Bezafibrate	99	±	12	110	±	8
Bisoprolol	98	±	8	112	±	4
Bupropion	99	±	9	113	±	2
Buspirone	73	±	4	102	±	6
Carazolol	101	±	4	106	±	7
Carbamazepine	101	±	5	115	±	7
Carbamazepine epoxide	97	±	8	116	±	15
Carboxine	91	±	24	106	±	12
Carfentrazone-ethyl	102	±	5	116	±	3
Celecoxib	72	±	6	83	±	8
Celecoxib-d7	80	±	12	72	±	5
Chloramphenicol	112	±	23	166	±	123
Cilazapril	90	±	3	109	±	2
Citalopram	94	±	9	118	±	8
Clarithromycin	32	±	6	13	±	10
Clarithromycin-d3	41	±	10	15	±	12
Clodinafop-propargyl	93	±	7	139	±	15
Clofibric acid	94	±	9	112	±	20

Clopidogrel	81	±	8	96	±	7
Clothianidin	105	±	16	107	±	22
Clozapine	78	±	9	99	±	7
Cocaine	97	±	5	118	±	2
Cocaine-d3	114	±	3	128	±	7
Cyclouron	92	±	5	113	±	9
Cycloxyidim	101	±	4	106	±	4
Cymoxanil	0	±	0	131	±	8
Cyromazine	0	±	0	127	±	12
Diazepam	102	±	14	107	±	0
Diazepam-d6	113	±	14	104	±	10
Diclofenac	97	±	11	125	±	2
Diflubenzuron	87	±	9	102	±	1
Dimethametryn	90	±	4	108	±	9
Dimethomorph	98	±	9	104	±	5
Diphenhydramine	97	±	4	123	±	9
Enalapril	91	±	20	102	±	4
Famoxadone	70	±	5	82	±	8
Fenoxaprop-ethyl	37	±	7	44	±	7
Fenuron	96	±	3	115	±	3
Flufenoxuron	0	±	0	0	±	0
Fluocinonide	63	±	7	101	±	24
Fluoxetine	88	±	10	120	±	9
Fluoxetine-d6	116	±	3	177	±	24
Flurbiprofen	89	±	43	116	±	33
Flurochloridone	99	±	16	110	±	35
Flutamide	92	±	4	106	±	8
Flutolanil	99	±	6	107	±	7
Fuberidazole	101	±	7	114	±	13
Hydrochlorothiazide	99	±	34	121	±	8
Imidacloprid	102	±	13	111	±	16
Indomethacin	0	±	0	0	±	0
Isocarbamid	97	±	8	115	±	9
Isradipine	78	±	5	109	±	28
Josamycin	0	±	0	0	±	0
Ketamine	99	±	5	114	±	4
Ketamine-d4	112	±	5	105	±	2
Ketoconazole	0	±	0	0	±	0
Ketoprofen	15	±	4	21	±	8
Ketotifen	96	±	4	108	±	3
Levamisole	98	±	5	114	±	7
Levocabastine	100	±	12	122	±	16
Lidocaine	97	±	6	107	±	7
Lidocaine-d10	109	±	1	103	±	1
Lincomycin	97	±	1	114	±	3
Lorazepam	100	±	7	117	±	14

Lorazepam-d4	109	±	3	102	±	11
MDMA	102	±	5	115	±	8
Meclizine	1	±	0	10	±	4
Medroxyprogesterone	73	±	1	77	±	5
Mefenamic acid	102	±	6	118	±	13
Memantine	101	±	5	114	±	10
Mephedrone	99	±	7	117	±	3
Mephosfolan	96	±	3	111	±	7
Metformin	0	±	0	114	±	2
Methamphetamine	105	±	7	108	±	6
Methcathinone	105	±	14	127	±	4
Methedrone	98	±	5	114	±	10
Methylone-d3	113	±	5	110	±	11
Methylphenidate	101	±	8	127	±	4
Methylphenidate-d9	110	±	5	124	±	5
Metoprolol	95	±	7	105	±	1
Metoprolol-d7	115	±	13	109	±	8
Morphine	115	±	32	123	±	24
Morphine-d3	103	±	4	100	±	15
Nadolol	102	±	6	113	±	7
Nicotine	96	±	10	86	±	29
Nicotine-d4	108	±	10	85	±	35
Nifedipine	102	±	22	94	±	31
Nifedipine-d4	146	±	93	93	±	45
Nitenpyram	95	±	6	120	±	7
Nordiazepam	100	±	10	115	±	9
Norethisterone	90	±	17	116	±	12
Nortriptyline	91	±	5	125	±	7
Nortriptyline-d3	118	±	6	162	±	12
Orphenadrine	91	±	5	116	±	4
Oxamyl	98	±	9	136	±	4
Oxycarboxin	106	±	5	123	±	15
Oxycodone	99	±	17	115	±	7
Picoxystrobin	85	±	6	99	±	10
Piperophos	70	±	4	81	±	9
Pirenzipine	98	±	8	108	±	10
Pretilachlor	80	±	4	92	±	4
Prodiamine	57	±	7	69	±	16
Prometon	97	±	9	112	±	10
Prometryn	96	±	5	109	±	3
Propamocarb	96	±	13	114	±	3
Propanolol	104	±	3	108	±	7
Propazine	96	±	4	113	±	7
Pymetrozine	89	±	7	122	±	6
Pyracarbolid	98	±	6	114	±	2
Pyraclostrobin	66	±	2	74	±	3

Pyraflufen-ethyl	51	±	5	62	±	11
Risperidone	75	±	3	93	±	15
Risperidone-d4	88	±	1	91	±	6
Rizatriptan	92	±	6	108	±	8
Ronidazole	96	±	8	121	±	2
Roxithromycin	5	±	2	2	±	3
Salbutamol	91	±	3	107	±	7
Salicylic acid	115	±	5	101	±	11
Simazine	100	±	4	119	±	13
Spinosyn A	0	±	0	0	±	0
Spinosyn D	0	±	0	0	±	0
Spiramycin	0	±	0	0	±	0
Sulfadimethoxine	104	±	9	110	±	4
Sulfamerazine	99	±	25	97	±	28
Sulfamethazine	105	±	14	98	±	2
Sulfamethazine-d4	117	±	7	95	±	6
Sulfamethoxazole	104	±	6	117	±	12
Sulfamonomethoxine	104	±	12	113	±	19
Sulfapyridine	98	±	6	110	±	4
Sulfathiazole	97	±	11	107	±	6
Sulfisoxazole	90	±	9	120	±	10
Tacrine	101	±	7	115	±	3
Tamsulosin	96	±	5	114	±	8
Temazepam	96	±	9	106	±	6
Temazepam-d5	109	±	2	102	±	3
Terbutryn	91	±	4	110	±	6
Terfenadine	0	±	0	0	±	0
Thiacloprid	100	±	6	111	±	10
Thiamethoxam	105	±	4	106	±	4
Thiamethoxam-d3	126	±	30	91	±	25
Thiazopyr	75	±	2	82	±	6
Timolol	97	±	4	117	±	8
Tramadol	99	±	7	113	±	4
Tramadol-13C1, d3	108	±	6	107	±	3
Trimethoprim	99	±	8	110	±	8
Trimethoprim-d3	111	±	2	107	±	7
Valsartan	102	±	10	104	±	32
Venlafaxine	99	±	7	112	±	10
Venlafaxine-d6	111	±	7	106	±	10
Verapamil	58	±	5	61	±	8
Verapamil-d3	71	±	2	67	±	10
Warfarin	104	±	9	111	±	11
Ziprasidone	22	±	4	57	±	7
**Average (all)**	**86**	**±**	**31**	**99**	**±**	**35**

Table S3. Method performance metrics for 113 compounds using the direct injection LC-MS/MS method in freshwater matrix according to ICH Harmonised Tripartite Guideline, Validation of Analytical Procedures: Text and Methodology, 2005.

**Analyte**	Range tested	Linearity	Peak Area Imprecision (RSD%), n=6		Matrix Effect (%), n=6		LLOD	LLOQ
	ng/L	R^2 (N>5)	250 ng/L	2500 ng/L	250 ng/L	2500 ng/L	ng/L	ng/L
Pharmaceuticals (n=68)								
Alprazolam	10-10000	0.995	11	6	-4	5	1.7	5.2
Amlodipine	10-10000	0.999	27	8	515	227	1.3	3.9
Amitriptyline	50-10000	0.974	21	6	107	56	15.7	47.0
Antipyrine	10-10000	1.000	4	6	-4	3	1.1	3.4
Atorvastatin	10-10000	0.997	5	6	32	28	1.5	4.4
Benzatropine	10-10000	0.997	21	6	146	98	1.5	4.5
Betaxolol	10-10000	0.998	7	5	12	5	1.8	5.4
Bezafibrate	10-10000	0.995	9	3	0	1	1.7	5.1
Bisoprolol	10-10000	0.999	5	3	5	6	1.2	3.6
Bupropion	10-10000	0.999	4	3	-5	-5	1.2	3.7
Buspirone	10-10000	0.999	9	7	-20	-16	1.3	4.0
Carazolol	10-10000	0.999	4	5	7	4	1.2	3.7
Carbamazapine	10-10000	0.991	5	3	1	3	2.1	6.2
Carbamazepine epoxide	10-10000	0.996	1	4	5	5	1.6	4.8
Carboxine	10-10000	0.997	1	4	-1	2	1.5	4.5
Celecoxib	10-10000	0.998	12	4	-8	1	1.8	5.5
Chloramphenicol	10-10000	0.985	36	6	-20	-1	2.5	7.5
Cilazapril	10-10000	0.994	4	3	3	2	1.8	5.4
Citalopram	10-10000	0.997	6	4	47	21	1.5	4.5
Clofibric acid	100-10000	0.982	19	4	8	5	2.7	8.2
Clopidogrel	10-10000	1.000	3	5	-1	-2	1.1	3.4
Clothianidin	50-10000	0.996	88	4	72	6	2.4	7.1
Diazepam	10-10000	0.999	9	5	-4	4	1.7	5.0
Diclofenac	50-10000	0.998	2	2	0	1	1.4	4.2
Diphenhydramine	10-10000	1.000	3	3	27	13	1.1	3.4
Fluocinonide	100-10000	0.976	43	6	9	-1	4.5	13.5
Fluoxetine	50-10000	0.995	24	10	288	126	3.2	9.7
Flutamide	10-10000	0.999	3	4	0	5	1.2	3.7
Haloperidol	250-10000	0.994	9	4	19	12	48.2	160.7
Hydrochlorothiazide	50-10000	0.987	30	6	-11	-16	2.7	8.0
Isradipine	100-10000	0.991	38	8	8	10	2.2	6.7
Ketotifen	10-10000	0.999	3	5	55	29	1.3	3.9
Levocabastine	250-10000	0.989	6	3	-4	1	2.2	6.6
Lidocaine	50-10000	0.997	4	3	1	-1	2.6	7.8
Lincomycin	10-10000	1.000	2	3	-5	-1	1.2	3.5
Medroxyprogesterone	10-10000	0.990	10	8	-10	-3	2.1	6.3
Mefenamic acid	50-10000	0.999	9	4	21	7	1.3	4.0
Memantine	10-10000	0.995	3	4	10	7	1.7	5.0
Methylphenidate	10-10000	0.999	3	4	-28	-29	1.8	5.3

Metoprolol	50-10000	0.983	8	4	11	2	10.0	30.1
Morphine	250-10000	0.996	13	3	-3	-16	3.2	9.7
Nadolol	10-10000	0.999	4	3	1	-1	1.2	3.6
Nortriptyline	50-10000	0.998	19	2	95	57	2.2	6.6
Orphenadrine	10-10000	1.000	7	6	49	24	1.1	3.4
Oxazepam	50-10000	0.992	10	4	4	1	2.1	6.4
Oxycodone	10-10000	0.999	2	3	-3	-2	1.3	3.9
Pirenzipine	10-10000	0.996	6	3	0	6	1.6	4.7
Propranolol	10-10000	0.999	4	3	6	1	1.3	3.9
Salbutamol	10-10000	0.989	3	4	6	12	2.3	6.8
Sulfadimethoxine	10-10000	0.996	3	4	19	12	1.6	4.8
Sulfamethaxole	10-10000	0.991	7	5	21	10	2.0	6.0
Sulfamethazine	50-10000	0.991	4	3	18	19	5.2	15.5
Sulfamonomethoxine	10-10000	0.998	10	5	21	18	1.5	4.4
Sulfapyridine	10-5000	0.993	4	3	15	21	1.9	5.7
Sulfathiazole	10-10000	0.991	2	2	6	16	2.1	6.2
Sulfisoxazole	10-10000	0.996	6	4	10	7	1.6	4.7
Tacrine	10-10000	0.999	4	4	8	5	1.3	3.8
Temazepam	50-10000	0.999	3	5	1	4	1.9	5.8
Thiamethoxam	10-10000	0.997	7	3	7	9	2.3	6.8
Timolol	10-10000	0.999	2	5	1	4	1.2	3.7
Tramadol	50-10000	0.988	3	3	3	3	7.6	22.8
Trimethoprim	10-10000	0.995	2	3	12	14	3.3	9.8
Valsartan	10-10000	0.999	14	4	10	7	1.3	4.0
Venlafaxine	100-10000	0.992	5	8	1	3	6.3	18.9
Warfarin	10-10000	1.000	4	3	-8	1	1.1	3.3
Ziprasidone	10-10000	0.982	34	19	-53	-57	2.8	8.3
Illicit Drugs (n=10)								
4-Fluoromethacationone	10-10000	0.999	5	4	-1	-11	1.3	3.9
4-Methylethcathinone	10-10000	0.997	3	3	-1	-6	1.5	4.6
Bezoylecgonine	10-10000	0.996	3	4	14	21	3.0	8.9
Cocaine	10-10000	0.998	3	4	-19	-22	1.9	5.8
Ketamine	10-10000	0.999	2	5	37	5	1.5	4.6
MDMA	10-10000	0.999	4	3	5	4	1.8	5.4
Mephedrone	10-10000	0.999	3	3	-2	-5	1.2	3.6
Methamphetamine	10-10000	0.998	2	3	2	9	1.4	4.3
Methcathinone	10-10000	0.984	2	4	-10	-16	2.6	7.8
Methedrone	10-10000	0.990	5	2	-3	2	2.2	6.5
Pesticides (n=36)								
Acetamiprid	10-10000	0.997	1	3	9	11	1.6	4.7
Ametryn	10-10000	1.000	3	3	1	3	1.2	3.5
Atrazine	10-10000	1.000	3	2	2	0	1.1	3.3
Azoxystrobin	50-10000	1.000	4	4	2	4	1.2	3.6
Benoxacor	10-10000	0.982	19	12	10	-6	2.8	8.3
Bensulide	10-10000	0.980	36	6	-15	8	3.0	8.9
Cyclouron	50-10000	0.988	6	4	4	4	2.6	7.7

Cycloxyidim	100-10000	0.975	6	2	21	13	3.3	10.0
Cyromazine	50-10000	0.999	5	2	-27	-27	1.4	4.1
Diflubenzuron	10-10000	0.998	7	5	-10	3	1.4	4.2
Dimethametryn	10-10000	0.999	5	3	2	2	1.2	3.6
Famoxadone	10-10000	0.999	3	4	0	5	1.3	3.8
Fenoxaprop-ethyl	10-10000	0.981	19	13	-13	-12	2.9	8.6
Fenuron	10-10000	0.999	4	1	-11	-1	1.3	3.9
Flutolanil	10-10000	1.000	5	4	5	2	1.2	3.5
Imidacloprid	10-10000	0.996	4	5	16	10	1.6	4.7
Isocarbamid	10-10000	0.991	7	4	0	3	6.5	19.6
Mephosfolan	10-10000	0.999	4	3	0	5	1.2	3.7
Nitenpyram	10-10000	0.969	3	4	2	2	3.8	11.5
Oxamyl	10-10000	0.997	11	5	-18	-14	1.5	4.6
Oxycarboxin	10-10000	0.995	6	3	-5	-5	1.7	5.1
Picoxystrobin	10-10000	0.976	8	4	2	-2	3.3	9.9
Piperophos	10-10000	0.999	6	6	-2	4	1.3	4.0
Pretilachlor	10-10000	0.994	7	2	-2	0	1.8	5.4
Prometon	10-10000	0.999	6	4	4	4	1.2	3.6
Prometryn	10-10000	1.000	4	4	3	2	1.2	3.5
Propamocarb	10-10000	0.998	6	3	5	11	1.4	4.1
Propazine	10-10000	0.999	4	4	0	2	1.2	3.7
Pymetrozine	10-10000	0.998	4	1	-28	-19	1.3	4.0
Pyracarbolid	10-10000	0.997	3	3	0	1	1.5	4.6
Pyraclostrobin	10-10000	0.999	10	7	-3	5	1.2	3.7
Ronidazole	10-10000	0.998	5	2	5	11	1.4	4.2
Simazine	10-10000	0.995	4	3	-4	-2	1.7	5.2
Tamsulosin	10-10000	0.999	3	4	10	9	1.2	3.6
Terbutryn	10-10000	0.999	2	5	4	0	1.3	3.8
Thiacloprid	10-10000	0.998	7	4	7	6	1.4	4.1
Thiazopyr	10-10000	0.999	6	5	0	1	1.2	3.6

Table S4. CEC occurrence in ng/L in Campaign 1 at the R. Schwarzach (SZ), Germany. All concentrations as the average of n=3 replicates

**Site code**	**SZ1**	**SZ2**	**SZ3**	**SZ4**	**SZ5**	**SZ6**	**SZ7**	**SZ8**	**SZ9**	**SZ10**
Acetamiprid	22.4	13.4	20.4	15.3	15.4	13.4	14.4	14.7	29.1	17.5
Antipyrine	21.9	9.5	24.0	12.9	12.0	19.6	16.8	19.2	198.9	41.6
Bezafibrate	15.5	11.4	16.7	25.5	24.1	25.9	20.3	23.7	60.5	35.8
Bisprolol	16.7	15.2	17.8	16.4	16.4	17.7	16.1	17.2	102.4	29.6
Carbamazepine	112.5	61.2	115.5	86.8	80.4	95.3	91.3	84.9	278.0	119.8
Carbamazepine epoxide	5.4	<LLOQ	5.7	<LLOQ	<LLOQ	<LLOQ	<LLOQ	<LLOQ	33.9	9.9
Citalopram	20.4	14.4	21.2	20.6	20.9	15.4	17.2	18.7	105.3	30.5
Clopidogrel	<LLOQ	<LLOQ	<LLOQ	<LLOQ	<LLOQ	<LLOQ	<LLOQ	<LLOQ	5.5	<LLOQ
Diclofenac	173.1	94.9	176.3	143.8	131.4	146.7	135.6	141.6	759.5	233.1
Diphenhydramine	<LLOQ	<LLOQ	<LLOQ	<LLOQ	<LLOQ	<LLOQ	3.6	<LLOQ	47.4	8.3
Fenuron	30.3	43.0	38.8	30.8	43.0	28.8	28.9	60.9	25.8	42.7
Hydrochlorothiazide	283.2	135.3	245.7	167.7	144.1	213.8	230.2	161.3	962.5	298.6
Lidocaine	22.9	15.6	22.3	18.1	19.4	17.6	19.6	18.3	39.6	23.8
Methamphetamine	7.5	4.8	9.2	5.7	5.9	7.3	6.5	6.1	30.2	13.6
Metoprolol	31.1	<LLOQ	34.1	<LLOQ	<LLOQ	<LLOQ	30.3	<LLOQ	284.5	55.3
Morphine	12.8	10.4	38.7	12.5	14.1	17.6	11.8	11.3	9.9	19.3
Nortriptyline	<LLOQ	<LLOQ	<LLOQ	<LLOQ	<LLOQ	<LLOQ	<LLOQ	<LLOQ	<LLOQ	<LLOQ
Oxazepam	91.4	106.7	102.7	95.4	79.3	86.9	90.1	79.5	90.7	85.1
Propranolol	4.6	<LLOQ	<LLOQ	<LLOQ	5.6	5.0	4.1	6.6	12.8	8.7
Sulfamethoxazole	8.5	<LLOQ	9.6	<LLOQ	6.5	6.4	6.8	6.3	119.8	16.5
Sulfapyridine	6.3	<LLOQ	<LLOQ	<LLOQ	<LLOQ	<LLOQ	<LLOQ	<LLOQ	36.1	7.2
Terbutryn	16.9	11.6	16.6	13.8	15.4	14.4	17.3	13.3	31.5	19.7
Tramadol	32.2	<LLOQ	32.9	26.1	26.3	28.2	28.2	27.7	89.8	39.8
Trimethoprim	<LLOQ	<LLOQ	<LLOQ	<LLOQ	<LLOQ	<LLOQ	<LLOQ	<LLOQ	62.4	12.0
Valsartan	56.4	53.6	68.7	74.9	73.1	84.9	80.5	74.3	387.6	121.8
Venlafaxine	35.4	23.7	36.0	30.1	31.1	28.6	32.0	31.1	154.4	49.0

Table S5 CEC occurrence in ng/L in Campaign 2 at the R. Emsbach (EM), Germany. All concentrations as the average of n=3 replicates.

Site code	EM1	EM2	EM3	EM4	EM5	EM6	EM7	EM8	EM9	EM10	EM11	EM12
4MC	<LLOQ	<LLOQ	<LLOQ	<LLOQ	<LLOQ	<LLOQ	6.2	<LLOQ	<LLOQ	4.7	<LLOQ	<LLOQ
Acetamiprid				<LLOQ	<LLOQ		30.2	4.9	5.5	7.5	10.0	4.9
Amitriptyline							130.9					
Antipyrine	14.0	18.3	19.7	26.5	14.6	18.9	239.6	45.5	52.3	47.4	78.4	56.1
Atorvastatin	<LLOQ			<LLOQ			<LLOQ				<LLOQ	<LLOQ
Bezafibrate		<LLOQ				<LLOQ	161.2	20.8	24.1	26.6	36.3	29.6
Bisprolol	<LLOQ	<LLOQ	<LLOQ	<LLOQ	<LLOQ	<LLOQ	496.4	66.3	68.1	81.3	100.9	77.8
Carbamazepine					<LLOQ		604.3	76.1	85.9	84.6	137.1	92.8
Carbamazepine epoxide							206.0	25.9	30.4	25.6	41.0	27.2
Citalopram	<LLOQ	<LLOQ	<LLOQ	<LLOQ	4.9	7.6	335.5	41.2	41.7	51.5	61.9	43.5
Clopidogrel	<LLOQ	<LLOQ	<LLOQ	<LLOQ		<LLOQ	17.1	4.3	<LLOQ	<LLOQ	7.0	3.7
Cocaine	<LLOQ	<LLOQ	6.6	7.0	6.8	<LLOQ	<LLOQ	<LLOQ	<LLOQ	<LLOQ	<LLOQ	<LLOQ
Diclofenac	4.6	5.2	6.1	10.0	71.9	45.8	1704.5	283.5	318.3	296.8	427.6	300.2
Diphenhydramine			4.0	5.7	3.7	5.1	136.2	18.8	24.9	21.0	30.8	17.5
Fenuron	111.2	153.1	102.0	107.0	171.4	114.5	121.4	148.3	147.8	102.7	168.6	122.4
Hydrochlorothiazide	<LLOQ	<LLOQ	<LLOQ	<LLOQ	14.8	<LLOQ	1963.9	312.9	322.0	287.2	545.2	306.3
Lidocaine							44.2	<LLOQ	<LLOQ	<LLOQ	9.0	<LLOQ
MDMA							13.1				2.7	
Mefenamic Acid	9.5	6.5	6.2	9.4	7.4	9.0	16.6	9.0	6.3	10.1	7.7	11.1
Memantine	<LLOQ		<LLOQ	<LLOQ			18.3	<LLOQ	<LLOQ	<LLOQ	5.4	<LLOQ
Metoprolol							157.6		<LLOQ	<LLOQ	<LLOQ	<LLOQ
Oxazepam	<LLOQ	<LLOQ	<LLOQ	<LLOQ	<LLOQ		28.7	<LLOQ	<LLOQ	<LLOQ	6.9	<LLOQ
Oxycodone	6.3	4.7	4.3	7.2	5.5	<LLOQ	67.8	14.5	18.1	15.7	20.9	18.4
Propranolol	<LLOQ	<LLOQ			<LLOQ	<LLOQ	40.8	5.4	4.6	6.7	8.7	4.8
Sulfamethoxazole	<LLOQ				<LLOQ		104.0	6.7	10.6	6.5	13.1	9.1
Tramadol							143.9	<LLOQ	<LLOQ	<LLOQ	28.7	<LLOQ
Trimethoprim							96.8	<LLOQ	10.8	<LLOQ	14.1	<LLOQ
Valsartan							626.1	49.2	54.5	46.3	106.3	70.4
Venlafaxine							149.3	<LLOQ	<LLOQ	<LLOQ	22.9	<LLOQ

Table S6. CEC occurrence in ng/L in Campaign 3 in the Basel city region, Switzerland in the Rivers Rhine (RH), Birs (BR), Birsig (BS), Wiese (WS). All concentrations as the average of n=3 replicates.

Site code	RH1	RH2	RH3	RH4	RH5	RH6	RH7	RH8	RH9	RH10	BS1	RH6	BR1	BR2	RH10	WS1	WS2	WS3	RH4
Antipyrine	4.0	3.9	4.7	<LLOQ	5.4	3.7	3.9	4.7	4.1	4.8	<LLOQ	3.7	<LLOQ	<LLOQ	3.9	4.3	3.8	4.7	<LLOQ
Atrazine	<LLOQ	<LLOQ	<LLOQ	<LLOQ	<LLOQ	3.8	<LLOQ	<LLOQ	<LLOQ	<LLOQ	6.8	3.8	<LLOQ	<LLOQ	<LLOQ		<LLOQ		
Benzatropine			<LLOQ	5.0												6.3			5.0
Benzoylecgonine						<LLOQ	<LLOQ		<LLOQ		<LLOQ	<LLOQ	<LLOQ	<LLOQ	<LLOQ				
Bezafibrate	5.2	<LLOQ	<LLOQ	<LLOQ	<LLOQ	<LLOQ	<LLOQ		<LLOQ		<LLOQ	<LLOQ	<LLOQ	<LLOQ	<LLOQ		<LLOQ	<LLOQ	<LLOQ
Bisoprolol	5.6	4.4	4.5	4.6	3.7	6.2	3.8	<LLOQ	4.2	3.9	7.9	6.2	4.5	4.5	3.8	5.2	4.6	4.5	4.6
Bupropion											<LLOQ								
Buspirone				4.4		<LLOQ						<LLOQ				<LLOQ			4.4
Carbamazepine	<LLOQ	<LLOQ	<LLOQ	<LLOQ	<LLOQ	23.0	<LLOQ	<LLOQ	<LLOQ	<LLOQ	38.4	23.0	<LLOQ	<LLOQ	<LLOQ	<LLOQ	<LLOQ	<LLOQ	<LLOQ
CBZ epoxide		<LLOQ			<LLOQ	<LLOQ			<LLOQ		<LLOQ	<LLOQ	<LLOQ	<LLOQ					
Celecoxib	10.0	9.0		11.3	<LLOQ	<LLOQ	<LLOQ	13.5	<LLOQ	<LLOQ	<LLOQ	<LLOQ	<LLOQ	<LLOQ	<LLOQ	<LLOQ	<LLOQ	<LLOQ	11.3
Chloramphenicol	11.7	11.7	24.1	17.6		12.1	11.2	<LLOQ	8.8	3.8	24.3	12.1	13.8		11.2	<LLOQ	11.3	<LLOQ	17.6
Citalopram	<LLOQ	<LLOQ	<LLOQ	16.7	<LLOQ	11.0		<LLOQ	<LLOQ	<LLOQ	17.7	11.0	4.8	<LLOQ		6.0			16.7
Clopidogrel						<LLOQ					<LLOQ	<LLOQ							
Cocaine	8.7	2.9	6.2	8.5	6.6	7.6	8.9	9.7	5.8	6.0	6.9	7.6	8.8	8.9	8.9	<LLOQ	7.9	7.5	8.5
Diclofenac	19.7	18.8	19.2	14.8	31.6	140.3	24.7	21.5	24.8	23.4	252.4	140.3	27.1	24.9	24.7	17.9	21.4	27.2	14.8
Diphenhydramine	<LLOQ	<LLOQ	<LLOQ	13.2		4.3					3.6	4.3		<LLOQ		7.8			13.2
Fluoxetine				<LLOQ															<LLOQ
Hydrochlorothiazide	9.5	11.9	18.3	23.9	<LLOQ	76.4	23.4	8.2	14.8	16.4	100.7	76.4	14.6	19.2	23.4	26.4	18.0	16.4	23.9
Ketotifen				<LLOQ															<LLOQ
Levocabastine				<LLOQ		<LLOQ						<LLOQ				<LLOQ			<LLOQ
Lidocaine					<LLOQ	11.6	<LLOQ	<LLOQ	<LLOQ	<LLOQ	19.5	11.6	<LLOQ	<LLOQ	<LLOQ				
Memantine											<LLOQ								
Methamphetamine	<LLOQ	<LLOQ			<LLOQ	<LLOQ	7.5	<LLOQ	<LLOQ	<LLOQ	4.4	<LLOQ	<LLOQ		7.5		<LLOQ	<LLOQ	
Metoprolol						<LLOQ					<LLOQ	<LLOQ							
Nortriptyline				<LLOQ												<LLOQ			<LLOQ

Orphenadrine				4.7												<LLOQ			<LLOQ
Oxazepam	<LLOQ	<LLOQ	<LLOQ	<LLOQ	6.7	11.3	8.3	<LLOQ	<LLOQ	8.3	18.6	11.3	9.8	12.3	8.3	<LLOQ	<LLOQ	<LLOQ	<LLOQ
Oxycodone			<LLOQ			<LLOQ					<LLOQ	<LLOQ							
Propamocarb	<LLOQ	<LLOQ	5.4	<LLOQ	<LLOQ	5.7	<LLOQ	<LLOQ	<LLOQ	<LLOQ	4.1	5.7	<LLOQ		<LLOQ	<LLOQ	<LLOQ	<LLOQ	<LLOQ
Propranolol	<LLOQ	<LLOQ	<LLOQ	<LLOQ	<LLOQ	<LLOQ	<LLOQ	<LLOQ		<LLOQ	<LLOQ	<LLOQ		<LLOQ	<LLOQ		<LLOQ	<LLOQ	<LLOQ
Sulfamethazine						<LLOQ					<LLOQ	<LLOQ							
Sulfamethoxazole						7.8					7.8	7.8							
Sulfapyridine			<LLOQ		<LLOQ			<LLOQ	<LLOQ		<LLOQ					<LLOQ	<LLOQ		
Tacrine				11.6		1.9						1.9				6.0			11.6
Terbutryn	<LLOQ	<LLOQ	<LLOQ	<LLOQ	<LLOQ	<LLOQ	<LLOQ	<LLOQ	<LLOQ	<LLOQ	<LLOQ	<LLOQ	<LLOQ	<LLOQ	<LLOQ	<LLOQ	<LLOQ	<LLOQ	<LLOQ
Tramadol					<LLOQ	23.0	<LLOQ		<LLOQ	<LLOQ	34.8	23.0	<LLOQ	<LLOQ	<LLOQ				
Trimethoprim						<LLOQ		<LLOQ		<LLOQ	<LLOQ	<LLOQ							
Valsartan	22.4	10.6	22.7	17.1	21.1	47.9	17.7	16.5	18.2	14.8	124.0	47.9	10.9	15.5	17.7	21.2	21.8	9.5	17.1
Venlafaxine	<LLOQ			<LLOQ	<LLOQ	<LLOQ	<LLOQ	<LLOQ			<LLOQ	<LLOQ	<LLOQ	<LLOQ	<LLOQ		<LLOQ	<LLOQ	<LLOQ

Table S7. CEC concentrations in ng/L in WWTP influent and effluent grab samples from Campaign 2 (data represent the average of triplicate measurements)

Compound	Influent	Effluent
4-Methcathinone	171	4
Acetamiprid	<LLOQ	31
Amitriptyline	16	41
Antipyrine	370	140
Atorvastatin	246	4
Benzoylecgonine	40	
Bezafibrate	1359	232
Bisoprolol	464	333
Bupropion	15	13
Carbamazepine	454	621
Carbamazepine Epoxide	48	181
Citalopram	146	229
Clopidogrel	28	12
Cocaine	219	6
Diclofenac	4043	1867
Diphenhydramine	63	116
Fenuron	246	69
Hydrochlorothiazide	2143	2750
Lidocaine	20	32
MDMA	8	11
Mefenamic Acid	78	18
Memantine	37	24
Metoprolol	48	51
Oxazepam	21	19
Oxycodone	95	30
Propranolol	31	38
Sulfamethoxazole	127	83
Tramadol	31	78
Trimethoprim	20	45
Valsartan	1292	1275
Venlafaxine	62	72
Verapamil	12	<LLOQ

Table S8. Classification of all compounds detected in river water samples to support interpretation of Figures 2, 3 and 5

Classification	Compounds						
Analgesics + NSAIDs	Antipyrine	Celecoxib	Diclofenac	Mefenamic acid	Morphine	Oxycodone	Tramadol
Anesthetics	Lidocaine						
Antibiotics	Chloramphenicol	Sulfamethazine	Sulfamethoxazole	Sulfapyridine	Trimethoprim		
Anticholinergics	Benzatropine	Orphenadrine					
Anticoagulants	Clopidogrel						
Anticonvulsants	Carbamazepine	CBZ epoxide					
Antidementia	Memantine						
Antidepressants	Amitriptyline	Bupropion	Citalopram	Fluoxetine	Nortriptyline	Venlafaxine	
Antihistamines	Diphenhydramine	Ketotifen	Levocabastine				
Antihyperlipidemics	Bezafibrate						
Antihypertensives	Bisoprolol	Metoprolol	Propranolol	Valsartan			
Antipsychotic	Buspirone						
Benzodiazepines	Oxazepam						
Cholinergics	Tacrine						
Diuretics	Hydrochlorothiazide						
Illicit Drugs	Benzoylecgonine	Cocaine	MDMA	Methamphetamine			
Pesticides	Acetamiprid	Atrazine	Fenuron	Propamocarb	Terbutryn		

Table S9. Risk quotients (RQs) for CECs determined at impacted sites SZ9, EM7 and RH5

Compound	Calculated RQ and site			PNEC (ng/L) [reference]
	SZ9	EM7	RH5	
Acetamiprid	0.01	<0.01	<0.01	3740 [1]
Amitriptyline		0.17	<0.01	140 [1]
Antipyrine	0.01	0.00	<0.01	21100 [2]
Atrazine			0.01	600 [1]
Benzoylecgonine			<0.01	4900 [3]
Bezafibrate	0.26	0.09	0.01	230 [4]
Bisprolol	0.03	0.02	<0.01	3180
Buspirone			0.03	74 [1]
Carbamazepine	5.56	1.88	0.46	50 [1]
Carbamazepine epoxide	0.01	0.01	<0.01	2570 [2]
Celecoxib			<0.01	1100 [5]
Chloramphenicol			0.03	401 [2]
Citalopram	0.01	<0.01	<0.01	16000 [1]
Clopidogrel	0.01	<0.01	<0.01	620 [1]
Cocaine		<0.01	<0.01	4900 [3]
Diclofenac	15.19	5.86	2.81	50 [1]
Diphenhydramine	0.05	0.01	<0.01	990 [1]
Fenuron	0.02	0.01	<0.01	1450 [1]
Hydrochlorothiazide	0.11	0.04	0.01	8380 [1]
Levocabastine			0.02	170 [2]
Lidocaine	0.01	0.01	<0.01	4670 [1]
MDMA		0.01	<0.01	47600 [2]
Mefenamic Acid		<0.01	<0.01	1720 [4]
Memantine		<0.01	<0.01	1840 [2]
Methamphetamine	0.00		<0.01	9740 [1]
Metoprolol	0.03	0.02	<0.01	8600 [2]
Morphine	<0.01		<0.01	5380 [1]
Nortriptyline	0.02		<0.01	190 [1]
Oxazepam	0.25	0.10	0.03	370 [1]
Oxycodone		0.00	<0.01	8040 [1]
Propamocarb			<0.01	710000 [2]
Propranolol	0.26	0.07	0.04	50 [4]
Sulfamethaxole	4.44	0.68	0.29	27 [4]
Sulfamethazine			<0.01	1120 [2]
Sulfapyridine	0.02		<0.01	1830 [2]
Tacrine			<0.01	520 [1]
Terbutryn	0.48		0.03	65 [1]
Tramadol	0.01	0.01	<0.01	8650 [1]
Trimethoprim	0.39	0.44	0.03	160 [4]
Valsartan	<0.01	<0.01	<0.01	560000 [1]
Venlafaxine	4.06	4.03	0.25	38 [1]

[1] NORMAN Ecotoxicology Database — Lowest PNECs (verified in freshwater) https://www.norman-network.com/nds/ecotox/lowestPnecsIndex.php?checkSelect=1; [2] NORMAN Ecotoxicology Database — Lowest PNECs (QSAR predicted in freshwater) https://www.norman-network.com/nds/ecotox/lowestPnecsIndex.php?checkSelect=0; [3] van der Aa et al., Water Research, 47, 2013, 1848; [4] Rivera-Jaimes et al., Science of the Total Environment, 613-4, 2018, 1263 [5] Vestel et al., Environmental Toxicology and Chemistry, 35, 2015, 1201.
